# Supplementary material for: Characterisation of a Staphylococcus aureus Isolate Carrying Phage-Borne Enterotoxin E from a European Badger (Meles meles)
Source: Pathogens. 2023 May 12;12(5):704. doi: 10.3390/pathogens12050704 (PMC10220527; doi:10.3390/pathogens12050704)
Supplement: Supplementary file 1 [file pathogens-12-00704-s001.zip › Supplemental File 1 Target Genes Primer and Probes.pdf]

SUPPLEMENTAL FILE 1: TARGET GENES, PRIMERS AND PROBES

Nr.	Symbol	Synonymes	Alleles	Name / description of gene or gene product	probe	Probe sequence	Primer sequence
1.	***aacA-aphD***	-	-	bifunctional enzyme Aac/Aph, gentamicin resistance	aacA-aphD_10,4	AB096217.1[28286:28313]	AB096217.1[28367:28386:r]
2.	***aadD***	-	-	aminoglycoside adenyltransferase, tobramycin resistance	aadD_1,2_PM4	AB037420.1[788:815]	AB037420.1[854:874:r]
3.	***agrB***	-	*agrB-I*	accessory gene regulator B	agrB-I_11	AF026120.1[3:29]	AF026120.1[57:79:r]
		-	*agrB-II*		agrB-II_11	AB043554.1[824:852]	AB043554.1[853:871:r]
		-	*agrB-III*		agrB-III_11	AB043555.1[971:1000]	AB043555.1[1029:1051:r]
		-	*agrB-IV*		agrB-IV_11	AF288215.1[1200:1226]	AF288215.1[1255:1275:r]
4.	***agrC***	-	*agrC-I*	accessory gene regulator C	agrC-I	AB043554.1[1741:1767]	AB043554.1[1826:1846:r]
		-	*agrC-II*		agrC-II	AF001782.1[892:917]	AF001782.1[983:1005:r]
		-	*agrC-III*		agrC-III	AF001783.1[1051:1077]	AF001783.1[1086:1107:r]
		-	*agrC-IV*		agrC-IV	AF288215.1[1553:1580]	AF288215.1[2049:2069:r]
5.	***agrD***	-	*agrD-I*	accessory gene regulator D	agrD-I_11<br>agrD-I_12<br>agrD-I_13	AF026120.1[144:171]<br>AJ617706.1[599:626]<br>AF210055.1[599:625]	AF026120.1[175:196:r]
		-	*agrD-II*		agrD-II	AF001782.1[650:679]	AF001782.1[681:702:r]
		-	*agrD-III*		agrD-III	AB043555.1[1072:1101]	AB043555.1[1108:1124:r]
6.	***aphA3***	-	-	3'5'-aminoglycoside phosphotransferase, neo-/kanamycin resistance	aphA-3_18,3	U51474.1[1553:1578:r]	U51474.1[1654:1671]
7.	***arcA***	-	*arcA-SCC*	ACME-locus	hp_arcA_611	AE015929.1[102505:102530:r]	AE015929.1[102460:102479]
8.	***arcB***	-	*arcB-SCC*	ACME-locus: ornithincarbamoyltransferase	hp_arcB_611	AE015929.1[99281:99307:r]	AE015929.1[99256:99274]
9.	***arcC***	-	*arcC-SCC*	ACME-locus: carbamatkinase	hp_arcC_611	AE015929.1[98603:98631:r]	AE015929.1[98571:98590]
10.	***arcD***	-	*arcD-SCC*	ACME-locus: arginine/ornithine-antiporter	hp_arcD_611	AE015929.1[101412:101440:r]	AE015929.1[101381:101398]
11.	***aur***	-	-	aureolysin	hp_aur_613,<br>hp_aur_611	AC027136.7[28379:28408:r],<br>AC027136.7[28846:28874:r]	AC027136.7[28345:28365],<br>AC027136.7[28812:28831]
		-	*aur-MRSA252*		hp_aur_613,<br>hp_aur_612	AC027136.7[28379:28408:r],<br>AJ249166.1[1026:1053]	AC027136.7[28345:28365],<br>AJ249166.1[1067:1088:r]
12.	***bap***	-	-	surface protein involved in biofilm formation	hp_bap_611	AY220730.1[7832:7860]	AY220730.1[7869:7891:r]
	bbp	-	*bbp-COL*	bone sialoprotein-binding protein	hp_bbp_616,<br>hp_bbp_614	AJ005647.1[2220:2246],<br>AB246401.1[76:104]	AB246401.1[2256:2278:r],<br>AB246401.1[132:149:r]
		-	*bbp-H6606*		hp_bbp_611	AM076252.1[3:31]	AM076252.1[37:59:r]
		-	*bbp-MRSA252*		hp_bbp_613,<br>hp_bbp_614	AB246401.1[1671:1696],<br>AB246401.1[76:104]	AB246401.1[2373:2393:r],<br>AB246401.1[2256:2278:r],<br>AB246401.1[1720:1739:r],<br>AB246401.1[132:149:r]
		-	*bbp-MW2*		hp_bbp_616,<br>hp_bbp_614	AJ005647.1[2220:2246],<br>AB246401.1[76:104]	AB246401.1[2373:2393:r],<br>AB246401.1[132:149:r]

Nr.	Symbol	Synonymes	Alleles	Name / description of gene or gene product	probe	Probe sequence	Primer sequence
		-	*bbp-Mu50*		hp_bbp_617, hp_bbp_614	AM076243.1[295:323], AB246401.1[76:104]	AB246401.1[2256:2278:r], AB246401.1[132:149:r], AM076243.1[349:368:r]
		-	*bbp-RF122*		hp_bbp_612, hp_bbp_614	AJ938182.1[578264:578291], AB246401.1[76:104]	AB246401.1[2373:2393:r], AB246401.1[132:149:r], AJ938182.1[578305:578327:r]
13.	***blaI***	-	-	beta lactamase repressor (inhibitor)	hp_blaI_611	AB179623.1[12885:12911]	AB179623.1[12931:12952:r]
14.	***blaR***	-	*blaR-MRSA252*	beta-lactamase regulatory protein	hp_blaR_612, hp_blaR_611	AB179623.1[12517:12546], AB179623.1[11832:11859]	AB179623.1[11892:11912:r], AB179623.1[12560:12583:r]
		-	*blaR-Sepi*		hp_blaR_613, hp_blaR_611	AE015929.1[1662669:1662697:r], AB179623.1[11832:11859]	AE015929.1[1663304:1663323], AB179623.1[12560:12583:r]
15.	***blaZ***	-	-	beta-lactamase	hp_blaZ_611, blaZ_11	AB179623.1[10303:10331:r], AB179623.1[10813:10837:r]	AB179623.1[10264:10281], AB179623.1[10713:10735]
16.	***blaZ - SCCmec XI***			beta-lactamase from SCC*mec* XI	hp_01_blaZ_M10	FR823292.1[1453:1479:r]	FR823292.1[1429:1448]
17.	***capH***	-	*capH1*	capsular polysaccharide synthesis enzyme CapH of capsule types 1, 5, and 8	hp_capH1_611	U10927.2[19165:19192]	U10927.2[19210:19230:r]
		-	*capH5*		hp_capH5_611	AC069081.4[6478:6502]	AC069081.4[6518:6538:r]
		-	*capH8*		hp_capH8_611	AJ938182.1[120208:120236]	AJ938182.1[120239:120260:r]
18.	***capI***	-	*capI8*	capsular polysaccharide biosynthesis protein CapI	hp_capI8_612	AJ938182.1[121964:121993]	AJ938182.1[122007:122027:r]
19.	***capJ***	-	*capJ1*	O-antigen polymerase CapJ of capsule types 1, 5, and 8	hp_capJ1_611	U10927.2[21322:21350]	U10927.2[21367:21385:r]
		-	*capJ5*		hp_capJ5_612, hp_capJ5_611	AC069081.4[8701:8730:r], AC069081.4[8535:8563]	AC069081.4[8673:8694], AC069081.4[8573:8590:r]
		-	*capJ8*		hp_capJ8_611	AJ938182.1[122465:122495]	AJ938182.1[122504:122521:r]
20.	***capK***	-	*capK1*	capsular polysaccharide biosynthesis protein CapK of capsule types 1, 5, and 8	hp_capK1_611	U10927.2[22439:22466]	U10927.2[22490:22508:r]
		-	*capK5*		hp_capK5_611	AC069081.4[9893:9921]	AC069081.4[9933:9952:r]
		-	*capK8*		hp_capK8_611, hp_capK8_612	AJ938182.1[122685:122712], AJ938182.1[123625:123651]	AJ938182.1[123653:123675:r], AJ938182.1[122716:122740:r]
21.	***cat***	-	*cat-pC221*	chloramphenicol acetyltransferase	hp_cat_613	M64281.1[358:389]	M64281.1[403:426:r]
		-	*cat-pC223*		hp_cat_611	AF507977.1[17615:17642]	AF507977.1[17658:17682:r]
		-	*cat-pMC524*		hp_cat_612	AB080798.1[2826:2854]	AJ312056.2[587:605:r], AB080798.1[2860:2878:r]
		-	*cat-pSBK203R*		hp_cat_615	M58515.1[353:384]	M58515.1[407:431:r]
22.	***ccrA***	-	*ccrA-1*	cassette chromosome recombinase A,	hp_ccrA-1_611, hp_ccrA-1_612	AB033763.2[24367:24393], AB033763.2[24614:24641]	AB033763.2[24397:24416:r], AB033763.2[24649:24667:r]
		-	*ccrA-2*		hp_ccrA-2_612, hp_ccrA-2_611	AB063172.2[10457:10485], AB063172.2[9903:9931]	AB063172.2[9939:9955:r], AB063172.2[10489:10507:r]
		-	*ccrA-3*		hp_ccrA-3_612, hp_ccrA-3_611	AB014436.1[787:813], AB014436.1[254:279]	AB014436.1[283:300:r], AB014436.1[826:844:r]
		-	*ccrA-4*		hp_ccrA-4_611	AF411935.1[8536:8563]	AF411935.1[8803:8823:r]
23.	***"ccrAA"***	-	*ccrAA-85-2082*	hypothetical protein accompanying *ccrC*	hp_ccrAA_611	AB037671.1[61431:61460:r]	AB037671.1[61397:61418]
		-	*ccrAA-MRSAZH47*		hp_ccrAA_613, hp_ccrAA_612	AM292304.1[6991:7016], AM292304.1[6601:6626]	AM292304.1[6629:6648:r], AM292304.1[7020:7038:r]

Nr.	Symbol	Synonymes	Alleles	Name / description of gene or gene product	probe	Probe sequence	Primer sequence
24.	***ccrB***	-	*ccrB-1*	cassette chromosome recombinase B	hp_ccrB-1_612,<br>hp_ccrB-1_613,<br>hp_ccrB-1_611	AB033763.2[25387:25411],<br>AB033763.2[25216:25245],<br>AB063171.1[13751:13773]	AB033763.2[25251:25268:r],<br>AB033763.2[25425:25445:r]
		-	*ccrB-2*		hp_ccrB-2_611,<br>hp_ccrB-2_612	AB063172.2[12275:12302],<br>AB063172.2[12499:12528]	AB063172.2[12548:12567:r],<br>AB096217.1[15001:15022:r],<br>AB063172.2[12308:12331:r]
		-	*ccrB-3*		hp_ccrB-3_611,<br>hp_ccrB-3_612	AB014436.1[2110:2135],<br>AB014436.1[2215:2243]	AB014436.1[2265:2286:r],<br>AB014436.1[2160:2179:r]
		-	*ccrB-4*		hp_ccrB-4_611	AE015929.1[59605:59629:r]	AE015929.1[59457:59474]
25.	***ccrC***	-	-	cassette chromosome recombinase B,	hp_ccrC_611	AB037671.1[60667:60697:r]	AB037671.1[60643:60662]
26.	***cfr***	-	-	23S rRNA methyltransferase	hp_cfr_611	AJ249217.1[1048:1074]	AJ249217.1[1075:1093:r]
27.	***chp***	-	-	chemotaxis-inhibiting protein (CHIPS)	hp_chp_611,<br>hp_chp_612	AB033232.1[8395:8421:r],<br>AB033232.1[8142:8170:r]	AB033232.1[8106:8129],<br>AB033232.1[8353:8373]
28.	***clfA***	-	*clfA-COL*	clumping factor A	hp_clfA_612,<br>hp_clfA_611	AC074317.5[4999:5027:r],<br>AB245456.1[496:524]	AB245456.1[1578:1599:r],<br>AB245456.1[550:569:r]
			clfA-MRSA252		hp_clfA_613,<br>hp_clfA_611	AB245456.1[1551:1576],<br>AB245456.1[496:524]	AB245456.1[1578:1599:r],<br>AB245456.1[550:569:r]
			clfA-MW2		hp_clfA_611,<br>hp_clfA_614	AB245456.1[496:524],<br>AM075836.1[1128:1152]	AB245456.1[1578:1599:r],<br>AB245456.1[550:569:r]
29.	***clfB***	-	*clfB-COL*	clumping factor B	hp_clfB_611,<br>hp_clfB_612	AC027136.7[20622:20652:r],<br>AC027136.7[19674:19705:r]	AC027136.7[20592:20610],<br>AC027136.7[19626:19648]
			clfB-MRSA252		hp_clfB_611,<br>hp_clfB_613	AC027136.7[20622:20652:r],<br>AM075901.1[1069:1098]	AJ938182.1[2647686:2647709],<br>AC027136.7[20592:20610]
			clfB-RF122		hp_clfB_614,<br>hp_clfB_611	AJ938182.1[2647736:2647765:r],<br>AC027136.7[20622:20652:r]	AJ938182.1[2647686:2647709],<br>AC027136.7[20592:20610]
30.	***cna***	-	-	collagen-binding adhesin	hp_cna_611	AB266874.1[435:461]	AB266874.1[469:487:r]
31.	***coa***	-	-	coagulase	coa_consens_11	CP000046.1 [246925:246954]	CP000046.1[246967:246988]
32.	***corB***	*hl*	-	putative membrane protein	hl_11	BA000017.4 [963137:963165]	BA000017.4[963188:963206:r]
33.	***dcs-Q9XB68***	-	*Q9XB68-dcs*	hypothetical protein from SCCmec elements	hp_Q9XB68_611	AB033763.2[38065:38093:r]	AB033763.2[38014:38037]
34.	***dfrA***	-	-	dihydrofolate reductase type 1	dfrA_12<br>2,1-dfrA	AE017171.1 [2588:2614:r]<br>AB049452.1 [2076:2103]	AE017171.1[2494:2513:r]
35.	***ebh***	-	-	cell wall associated fibronectin-binding protein	hp_ebh-3prime_611	AJ938182.1[1406914:1406940:r]	AJ938182.1[1405560:1405579]
36.	***ebpS***	-	*ebpS*	cell surface elastin binding protein	hp_ebpS_614,<br>hp_ebpS_612,<br>hp_ebpS_613	AF400161.1[975:999],<br>AF400161.1[273:300],<br>AF400161.1[511:536]	AF400161.1[1013:1030:r],<br>AF400161.1[546:569:r],<br>AF400161.1[303:321:r]
			ebpS-01-1111 (aus CC45)		hp_ebpS_614,<br>hp_ebpS_611	AF400161.1[975:999],<br>AM075954.1[148:172]	AF400161.1[1013:1030:r],<br>AF400161.1[303:321:r]
37.	***edinA***	-	-	epidermal cell differentiation inhibitor precursor	edinA_11	M63917.1[460:489]	M63917.1[502:520:r]
38.	***edinB***	-	-	epidermal cell differentiation inhibitor B	edinB_11	AB057421.1[7445:7471]	AB057421.1[7482:7501:r]
39.	***edinC***	-	-	epidermal cell differentiation inhibitor C	edinC_11	AP003088.1[1810:1839:r]	AP003088.1[1755:1776]
40.	***eno***	-	-	enolase	hp_eno_611	AC074317.5[16708:16737:r]	AC074317.5[16665:16683]

Nr.	Symbol	Synonymes	Alleles	Name / description of gene or gene product	probe	Probe sequence	Primer sequence
41.	***"entX"***	SACOL1657	-	hypothetical enterotoxin homologue	entX_11	BA000017.4[1708858:1708886:r]	BA000017.4[1708835:1708855]
42.	***erm*(A)**	***ermA***	-	rRNA adenine N-6-methyltransferase, erythromycin/ clindamycin resistance	ermA_9,4	BA000017.4 [1762850:1762875]	BA000017.4[1762907:1762928]
43.	***erm*(B)**	***ermB***	-	erythromycin/clindamycin resistance	hp_ermB_611<br>hp_ermB_612	EF450709.1 [2405:2428]<br>EF450709.1 [2528:2557]	EF450709.1 [2443:2464:r]<br>EF450709.1 [2564:2584:r]
44.	***erm*(C)**	***ermC***	-	erythromycin/clindamycin resistance	ermC_8,1_PM4<br>ermC_8,2_PM4	AE002098.2[75789:75813],<br>AE002098.2[75854:75878]	AE002098.2[75909:75929:r]
45.	***etA***	-	-	exfoliative toxin serotype A	8,2-etA	AP001553.1[42317:42344]	AP001553.1[42387:42406:r]
46.	***etB***	-	-	exfoliative toxin serotype B	9,3-etB	AP003088.1[5389:5416]	AP003088.1[5438:5460:r]
47.	***etD***	-	-	exfoliative toxin D	etD_11	AB057421.1[5648:5677]	AB057421.1[5694:5715:r]
48.	***far1***	***fusB***	-	fusidic acid resistance (plasmid-borne)	far1_10	AY047358.1 [1787:1814]	AY047358.1[1818:1838:r]
49.	***fexA***	-	-	chloramphenicol/florfenicol exporter	hp_fexA_611	AJ549214.1[332:357]	AJ549214.1[364:382:r]
50.	***fib***	-	-	fibrinogen binding protein (19 kDa)	hp_fib_611,<br>hp_fib_612	AJ306909.1[483:507],<br>BX571856.1[1178081:1178105]	AJ306909.1[511:528:r]
51.	***fnbA***	-	*fnbA-COL*	fibronectin-binding protein A	hp_fnbA_615,<br>hp_fnbA_612	AJ629121.1[1767:1795],<br>AJ629121.1[983:1009]	AJ629121.1[1823:1843:r],<br>AJ629121.1[1031:1052:r]
			fnbA-MRSA252		hp_fnbA_615,<br>hp_fnbA_613	AJ629121.1[1767:1795],<br>AM076028.1[1027:1055]	AM076028.1[1061:1079:r],<br>AJ629121.1[1823:1843:r]
			fnbA-MW2		hp_fnbA_615,<br>hp_fnbA_611	AJ629121.1[1767:1795],<br>AM075994.1[630:657]	AM075994.1[672:693:r],<br>AJ629121.1[1823:1843:r]
			fnbA-RF122		hp_fnbA_615,<br>hp_fnbA_614	AJ629121.1[1767:1795],<br>AJ938182.1[2510055:2510084:r]	AJ629121.1[1823:1843:r],<br>AJ938182.1[2510030:2510048]
			fnbA-ST80		hp_fnbA_615	AJ629121.1[1767:1795]	AJ629121.1[1823:1843:r]
52.	***fnbB***	-	*fnbB-COL*	fibronectin-binding protein B	hp_fnbB_614,<br>hp_fnbB_616	AJ629122.1[991:1017],<br>AJ629122.1[1658:1688]	AJ629122.1[1041:1061:r],<br>AJ629122.1[1697:1714:r]
			fnbB-MW2		hp_fnbB_613,<br>hp_fnbB_616	AM076068.1[854:883],<br>AJ629122.1[1658:1688]	AM076068.1[893:912:r]
			fnbB-Mu50		hp_fnbB_611,<br>hp_fnbB_616	AM076047.1[866:895],<br>AJ629122.1[1658:1688]	AM076047.1[795:817:r],<br>AJ629122.1[1697:1714:r],<br>AM076047.1[914:934:r]
			fnbB-ST15		hp_fnbB_611,<br>hp_fnbB_612	AM076047.1[866:895],<br>AM076087.1[758:783]	AM076057.1[920:940:r],<br>AM076047.1[795:817:r]
			fnbB-ST36		hp_fnbB_611	AM076047.1[866:895]	AM076047.1[795:817:r],<br>AM076079.1[914:933:r]
			fnbB-ST45-2		hp_fnbB_615	AM076078.1[866:893]	AM076047.1[795:817:r],<br>AM076078.1[900:920:r]
53.	***fosB***	-	*fosB*	metallothiol transferase	hp_fosB_611	AP009324.1[2460791:2460821]	AP009324.1[2460852:2460871:r]
			fosB-plasmid		hp_fosB_612	AP006717.1[448:478]	AP006717.1[508:527:r]
54.	***fusC***	**Q6GD50**	-	SCC-encoded protein associated with fusidic acid resistance	hp_Q6GD50_611	AF411935.1[423:452:r]	AF411935.1[372:390]
55.	***gapA***	-	-	glyceraldehyde 3-phosphate dehydrogenase, locus 1	hp_gapA-118704_PM5	AC074317.5[21403:21431:r]	AC074317.5[21373:21393]
56.	***hla***	-	-	haemolysin alpha	hla_11	AC027137.8[47755:47784]	AC027137.8[47798:47820:r]

Nr.	Symbol	Synonymes	Alleles	Name / description of gene or gene product	probe	Probe sequence	Primer sequence
57.	***hlb***	-	-	haemolysin beta	hp_hlb_611,<br>hp_hlb_612,<br>hp_hlb_613,<br>hlb_11<br>hlb_12	CP000046.1 [2063898:2063922],<br>BA000017.4 [2126171:2126196],<br>S72497.1[366:390],<br>CP000046.1 [2063880:2063906],<br>S72497.1[347:374]	CP000046.1[2063925:2063944:r]
58.	***hld***	-	-	haemolysin delta	hld_11	AB043554.1[196:220:r]	AB043554.1[153:175]
59.	***hlgA***	-	-	haemolysin gamma, component A	hlgA_11	BA000017.4 [2549687:2549713]	BA000017.4[2549714:2549735:r]
60.	***hlIII***	SAV2170,<br>SACOL2160	*hlIII- other than RF122*	putative membrane protein	hl-III_11	AB078343.1[539:567]	AB078343.1[998:1019:r]
			hlIII-consensus		hp_hlIII_611	AB078343.1[951:976]	AB078343.1[590:612:r]
61.	***hsdS1***	-	*hsdS1-RF122*	type I site-specific deoxyribonuclease subunit, 1st locus	hp_hsdS-RF122-1_611	AJ938182.1[317663:317689]	AJ938182.1[317702:317723:r]
62.	***hsdS2***	-	*hsdS2-ST5+ST8*	type I site-specific deoxyribonuclease subunit, 2nd locus	hp_hsdS-COL-1_611	AP009324.1[478662:478690]	AP009324.1[478701:478719:r]
			hsdS2-MW2+476		hp_hsdS-MW2-1_611	BA000033.2[442980:443006]	BA000033.2[443035:443058:r],<br>AJ938182.1[1812895:1812912]
			hsdS2-RF122		hp_hsdS-RF122-2_611	AJ938182.1[422326:422351]	AJ938182.1[422376:422398:r]
			hsdS2-MRSA252		hp_hsdS-MRSA252-1_611	BX571856.1[463045:463073]	BX571856.1[463099:463120:r]
63.	***hsdS3***	-	*hsdS3-AllOtherThanRF122+252*	type I site-specific deoxyribonuclease subunit, 3rd locus	hp_hsdS-CC25_611	DQ309452.1[57:85]	AJ938182.1[1812895:1812912]
			hsdS3-ST8+ST1+RF122		hp_hsdS-COL-2_611	AJ938182.1[1811960:1811988:r]	AJ938182.1[1812895:1812912]
			hsdS3-Mu50+N315		hp_hsdS-Mu50-2_611	AP009324.1[1937287:1937313:r]	AP009324.1[1937243:1937264]
			hsdS3-CC51+252		hp_hsdS-CC51_611	BX571856.1[1983689:1983715:r]	BX571856.1[1983667:1983686]
			hsdS3-MRSA252		hp_hsdS-MRSA252-2_611	BX571856.1[1983034:1983063:r]	BX571856.1[1983667:1983686]
64.	***hsdSx***	-	*hsdSx-CC25*	type I site-specific deoxyribonuclease subunit, unknown locus	hp_hsdS-CC25_612	AP009324.1[1938268:1938295:r]	AJ938182.1[1811926:1811943]
			hsdSx-CC15		hp_hsdS-CC15_611	DQ309450.1[976:1000]	DQ309450.1[1009:1031:r]
			hsdSx-etd		hp_hsdS-etd_611	AB057421.1[2572:2598:r]	AB057421.1[2543:2565]
65.	***hysA1/2***	-	*hysA1-MRSA252*	hyaluronate lyase, first / second locus	hp_hysA_613	BX571856.1[1975471:1975495]	AC078831.10[18640:18660:r],<br>AJ938182.1[1803841:1803863:r]
			hysA1-MRSA252+RF122 and hysA2-all		hp_hysA_614	AC078831.10[18606:18636]	
			hysA1-MRSA252+RF122 and hysA2-COL+USA300		hp_hysA_615	AJ938182.1[1803808:1803836]	
66.	***hysA2***	-	*hysA2-AllOtherThan252*	hyaluronate lyase, second locus	hp_hysA_611	AC078831.10[17303:17329]	AC078831.10[17230:17253:r],<br>AC078831.10[17343:17361:r],<br>AC078831.10[18640:18660:r]
			hysA2-COL+USA300+NCTC		hp_hysA_617	AC078831.10[17198:17228]	
			hysA2-AllOtherThan COL+USA300+NCTC		hp_hysA_616	AJ938182.1[2230577:2230606]	
			hysA2-OtherThan COL+USA300+NCTC		hp_hysA_618	AP009324.1[2345045:2345075]	
			hysA2-MRSA252		hp_hysA_612	AY442448.1[26:54],	
67.	***icaA***	-	-	intercellular adhesion protein A	hp_icaA_611	AF086783.1[3376:3401]	AF086783.1[3422:3442:r]
68.	***icaC***	-	-	intercellular adhesion protein C	hp_icaC_611	AF086783.1[5365:5394]	AF086783.1[5402:5420:r]
69.	***icaD***	-	-	biofilm PIA synthesis protein D	hp_icaD_611	AF086783.1[3681:3710]	AF086783.1[3738:3760:r]

Nr.	Symbol	Synonymes	Alleles	Name / description of gene or gene product	probe	Probe sequence	Primer sequence
70.	***isaB***		*isaB*	immunodominant antigen B	hp_isaB_611	AC027136.7[30242:30266:r]	AC027136.7[30213:30231]
			isaB-MRSA252		hp_isaB_612	BX571856.1[2813801:2813828:r]	BX571856.1[2813775:2813793]
71.	***isdA***	-	*isdA*	transferrin-binding protein	hp_isdA_611,<br>hp_isdA_612,<br>hp_isdA_614	AB042826.1[872:896],<br>AB042826.1[991:1015],<br>AJ938182.1[1075980:1076008:r]	AB042826.1[1037:1059:r],<br>AB042826.1[901:919:r],<br>AJ938182.1[1075946:1075968]
			isdA-MRSA252		hp_isdA_611,<br>hp_isdA_612	AB042826.1[872:896],<br>AB042826.1[991:1015]	AB042826.1[1037:1059:r],<br>AB042826.1[901:919:r]
72.	***katA***	-	-	katalase A	katA_11	BA000017.4 [1409422:1409446]	BA000017.4[1409453:1409473:r]
73.	***kdpA***	-	*kdpA-SCC*	potassium-translocating ATPase A, chain 2	hp_kdpA-SCC_612,<br>hp_kdpA-SCC_611	AB033232.1[25:53],<br>AP006716.1[38978:39004]	AB033232.1[63:83:r],<br>AP006716.1[39037:39057:r]
74.	***kdpB***	-	*kdpB-SCC*	potassium-transporting ATPase B, chain 1	hp_kdpB-SCC_611	AB033232.1[1908:1935]	AB033232.1[1948:1967:r]
75.	***kdpC***	-	*kdpC-SCC*	potassium-translocating ATPase C, chain 2	hp_kdpC-SCC_611,<br>hp_kdpC-SCC_612	AB033232.1[3099:3125],<br>AB033232.1[3206:3232:r]	AB033232.1[3133:3151:r],<br>AB033232.1[3182:3202]
76.	***kdpD***	-	*kdpD-SCC*	sensor kinase protein	hp_kdpD-SCC_611	AP006716.1[37752:37779:r]	AP006716.1[37713:37731]
77.	***kdpE***	-	*kdpE-SCC*	KDP operon transcriptional regulatory protein	hp_kdpE-SCC_611	AP006716.1[35126:35151:r]	AP006716.1[35099:35117]
78.	***linA***	-	-	lincosamid-nucleotidyltransferase	linA_19,2<br>linA_19,3	AM184101.1[2144:2168:r],<br>AM184101.1[2216:2240:r]	AM184101.1[2037:2057],<br>AM184101.1[2053:2070]
79.	***lmrP***	-	-	hypothetical protein, similar to integral membrane protein LmrP	hp_lmrP_613,<br>hp_lmrP_611	AC069081.4[27540:27566],<br>AC069081.4[26853:26878]	AC069081.4[26886:26903:r],<br>AC069081.4[27570:27590:r]
			lmrP-RF122		hp_lmrP_612,<br>hp_lmrP_614	AJ938182.1[140620:140646],<br>AJ938182.1[141308:141333]	AJ938182.1[140655:140672:r],<br>AJ938182.1[141338:141358:r]
80.	***lukD***	-	-	leukocidin D component	lukD_11	AB055623.1[2367:2396]	AB055623.1[2421:2441:r]
81.	***lukE***	-	-	leukocidin E component	lukE_11	AB055623.1[1159:1183]	AB055623.1[1207:1226:r]
82.	***lukF-hlg***	-	-	haemolysin gamma, component B	lukF-10	BA000017.4[2552175:2552200]	BA000017.4[2552204:2552221:r]
83.	***lukF-PV***	-	-	Panton Valentine leukocidin F component	lukF-PV_10	AB006796.1[2256:2284]	AB006796.1[2295:2316:r]
84.	***lukF-PV83***	-	-	F component from hypothetical leukocidin from ruminants	lukF-PV-P83_11	AB044554.1[42010:42037]	AB044554.1[42053:42070:r]
85.	***lukM***	-	-	S component from hypothetical leukocidin from ruminants	lukM_11	AB044554.1[40866:40893]	AB044554.1[40914:40932:r]
86.	***lukS-hlg***	-	*lukS*	haemolysin gamma, component C	lukS_10	BA000017.4 [2551185:2551209]	BA000017.4[2551213:2551233:r]
			lukS-ST45		hp_lukS-ST45_611	EF672356.1[663:686]	EF672356.1[690:710:r]<br>EF672356.1[690:711:r]
87.	***lukS-PV***	-	-	Panton Valentine leukocidin S component	lukS-PV_20	AB006796.1[1628:1656]	AB006796.1[1679:1699:r]
88.	***"lukX"***	SAV2004, *lukG, lukA*	-	leukocidin/haemolysin toxin family protein	lukX_11	BA000017.4[2127329:2127353:r]	BA000017.4[2127284:2127306]
89.	***"lukY"***	SAV2005, *lukH, lukB*	*lukY*	leukocidin/haemolysin toxin family protein	lukY-var1_11	BA000017.4[2129068:2129097:r]	BA000017.4[2129030:2129050]
			lukY-MRSA252		lukY-var2_11	BX571856.1[2171414:2171443:r]	
90.	***map***	*eap*	*map-7*	Major histocompatibility complex class II analogue protein (=Extracellular adherence protein, *eap*)	hp_map_613	AJ132841.1[368:395]	AJ132841.1[420:438:r],<br>AJ223806.1[279:298:r]
			map-COL		hp_map_611	AJ223806.1[226:254]	
			map-IPOP2		hp_map_612	AJ245439.1[361:385]	

Nr.	Symbol	Synonymes	Alleles	Name / description of gene or gene product	probe	Probe sequence	Primer sequence
91.	***mecA***	-	-	Modified penicillin binding protein 2, beta-lactam resistance defining MRSA	mecA_1,4<br>mecA_11	CP000046.1 [39915:39942:r],<br>CP000046.1 [40041:40068:r]	CP000046.1[39857:39876:r],<br>CP000046.1[40007:40025]
92.	***mecC***			Alternative *mec* gene from SCC*mec* XI	17_mecA_hp<br>25_mecA_hp	FR823292.1[3006:3032:r]<br>FR823292.1[2830:2862:r]	FR823292.1[2968:2988:r]<br>FR823292.1[2764:2788:r]
93.	***mecI***	-	-	methicillin-resistance regulatory protein	hp_mecI_611	AB037671.1[22423:22452:r]	AB037671.1[22395:22416]
94.	***mecR1***	*mecR*	-	signal transducer protein MecR1	hp_mecR_611,<br>hp_mecR_612	AB033763.2[31157:31186:r],<br>AB037671.1[22874:22900:r]	AB033763.2[31133:31154],<br>AB037671.1[22840:22859]
95.	***mefA***	-	-	macrolide efflux protein A	hp_mefA_611,<br>hp_mefA_612	AB011259.1[536:563],<br>AB011259.1[1045:1072]	AB011259.1[570:588:r],<br>AB011259.1[1078:1099:r]
96.	***merA***	-	-	mercury-reductase	hp_merA_611	AB037671.1[39354:39382:r]	AB037671.1[39315:39334]
97.	***merB***	-	-	mercuric resistance operon regulatory protein	hp_merB_611	AB037671.1[38021:38046:r]	AB037671.1[38000:38018]
98.	***mph*(BM)**	*mpbBM*,<br>*mphBM*	-	probable lysylphosphatidylglycerol synthetase	hp_mpbBM_611,<br>hp_mpbBM_612	AB013298.1[2664:2693],<br>AB013298.1[2896:2924]	AB013298.1[2700:2720:r],<br>AB013298.1[2929:2947:r]
99.	***mprF***	-	-	energy-dependent efflux of erythromycin	hp_mprF_612,<br>hp_mprF_611	AP009324.1[1443571:1443601],<br>AB043507.1[2677:2707]	AB043507.1[2708:2731:r]
100.	***msr*(A)**	*msrA*	-	mercuric resistance operon regulatory protein	msrA_15,3	AB013298.1[1525:1552]	AB013298.1[1614:1635:r]
101.	***mupR***	*mupA*	-	mupirocin resistance protein	mupR_13,2	X75439.1[1504:1531]	X75439.1[1623:1642:r]
102.	***nuc1***	-	-	thermostable extracellular nuclease	hp_nuc1_611	AJ938182.1[825403:825429]	AJ938182.1[825445:825462:r]
103.	***ORF CM14***	SAB0026	-	enterotoxin-like protein ORF CM14	hp_entCM14_611,<br>hp_entCM14_612	AJ938182.1[37154:37182],<br>AJ938182.1[37532:37557]	AJ938182.1[37591:37610:r],<br>AJ938182.1[37203:37221:r]
104.	***pls-SCC***	-	-	plasmin-sensitive surface protein	hp_plsSCC_611	AB033763.2[15685:15709:r]	AB033763.2[15641:15661]
105.	***Q2FXC0***	-	-	hypothetical protein, located next to serine protease operon	hp_Q2FXC0_611	AP009351.1[1905375:1905400]	AP009351.1[1905410:1905429:r]
106.	***Q2YUB3***	-	-	unspecific efflux/transporter	hp_Q2YUB3_611	AJ938182.1[2026944:2026969:r]	AJ938182.1[2026920:2026937]
107.	***Q7A4X2***	-	-	hypothetical protein	hp_Q7A4X2_611	AJ938182.1[1837306:1837335]	AJ938182.1[1837354:1837376:r]
108.	***qacA***	-	-	quaternary ammonium compound resistance protein A	hp_qacA_611	AB255366.1[19119:19147]	AB255366.1[20504:20526:r],<br>AB255366.1[20475:20498:r],<br>AB255366.1[19153:19173:r]
109.	***qacC***	-	*qacC*	quaternary ammonium compound resistance protein C	hp_qacC_611	AB125342.1[2382:2411]	AB125342.1[2431:2450:r]
			qacC-SA5		hp_qacC_613	U81980.1[2017:2043]	U81980.1[2065:2086:r]
			qacC-ST94		hp_qacC_615	Y16944.1[1622:1649]	Y16944.1[1692:1714:r]
			qacC-Ssap		hp_qacC_612	Y16945.1[1951:1981]	AE016833.1[8848:8869:r]
			qacC-equine		hp_qacC_614	AJ512814.1[1518:1545]	AJ512814.1[1567:1590:r]
110.	***rrn STAU***	-	-	Ribosomal sequence from *S. aureus* (genus-specific positive control)	s_aur_rrn_1_pm_PM4	BA000017.4[1999541:1999566:r]<br>BA000018.3[1921746:1921771:r]	BA000017.4[1999503:1999521:r]<br>BA000018.3[1921708:1921726:r]
111.	***saeR***	-	-	response regulator, sae locus	hp_saeR_611	AF129010.1[251:280]	AF129010.1[296:316:r]
112.	***saeS***	-	-	histidine protein kinase, sae locus	hp_saeS_611,<br>hp_saeS_612	AF129010.1[1229:1257],<br>AF129010.1[1729:1758]	AF129010.1[1283:1304:r],<br>AF129010.1[1770:1791:r]
113.	***sak***	-	-	staphylokinase	hp_sak_611,<br>sak_11	BA000017.4 [2086572:2086601:r]<br>BA000017.4 [2086418:2086443:r]	BA000017.4[2086553:2086571],<br>BA000017.4[2086376:2086395]
114.	***sarA***	-	-	staphylococcal accessory regulator A	hp_sarA_611,	CP000046.1 [700076:700102:r]	CP000046.1[700049:700066]

Nr.	Symbol	Synonymes	Alleles	Name / description of gene or gene product	probe	Probe sequence	Primer sequence
115.	***sasG***	-	*sasG-COL*	*Staphylococcus aureus* surface protein G	hp_sasG_613, hp_sasG_611	AP009324.1[2635299:2635327:r], AP009324.1[2635746:2635773:r]	AP009324.1[2635268:2635289], AP009324.1[2635703:2635725]
			sasG-MW2		hp_sasG_612, hp_sasG_611	BA000033.2[2573572:2573601:r], AP009324.1[2635746:2635773:r]	AP009324.1[2635703:2635725], BA000033.2[2573526:2573543]
116.	***sat***	-	-	streptothricin-acetyltransferase	sat-17,2 sat-17,3	U51474.1[393:421] U51474.1[429:456]	U51474.1[488:505:r]
117.	***sbi***	-	-	IgG-binding protein	sbi-var1_11, sbi-var1_12	CP000046.1 [2476904:2476929], CP000046.1 [2477142:2477171]	CP000046.1[2476963:2476982:r] CP000046.1[2477188:2477210:r]
118.	***scn***	-		Staphylococcal complement inhibitor (SCIN)	hp_scn_611	AF424783.1[41412:41440]	AF424783.1[41450:41470:r]
119.	***sdrC***	-	*sdrC-B1*	Ser-Asp rich fibrinogen-/bone sialoprotein-binding protein C	hp_sdrC_612, hp_sdrC_613	AM076155.1[1009:1036], AJ005645.1[679:705]	AJ938182.1[574454:574471:r], AM076155.1[1039:1061:r]
			sdrC-COL		hp_sdrC_611, hp_sdrC_613 hp_sdrC_615,	AJ005645.1[280:308], AJ005645.1[679:705] AJ005645.1[1288:1314],	AJ005645.1[322:341:r], AJ005645.1[726:744:r] AJ005645.1[1337:1355:r],
			sdrC-MRSA252		hp_sdrC_616, hp_sdrC_613	AJ938182.1[575014:575042], AJ005645.1[679:705]	AJ938182.1[574454:574471:r]
			sdrC-MW2		hp_sdrC_616, hp_sdrC_611, hp_sdrC_613	AJ938182.1[575014:575042], AJ005645.1[280:308], AJ005645.1[679:705]	AJ005645.1[322:341:r], AJ005645.1[1337:1355:r], AJ005645.1[726:744:r]
			sdrC-Mu50		hp_sdrC_614, hp_sdrC_611, hp_sdrC_613	AM076143.1[1099:1125], AJ005645.1[280:308], AJ005645.1[679:705]	AJ005645.1[322:341:r], AJ005645.1[1337:1355:r], AJ005645.1[726:744:r]
120.	***sdrD***	-	*sdrD-COL*	Ser-Asp rich fibrinogen-/bone sialoprotein-binding protein D	hp_sdrD_614, hp_sdrD_612	AJ005646.1[1866:1893], AJ005646.1[1207:1232]	AJ005646.1[1237:1258:r], AJ005646.1[1903:1924:r]
			sdrD-Mu50		hp_sdrD_614, hp_sdrD_613	AJ005646.1[1866:1893], AM076196.1[158:186]	AJ005646.1[1903:1924:r], AM076196.1[190:210:r]
			sdrD-other1		hp_sdrD_614, hp_sdrD_611	AJ005646.1[1866:1893], AM076206.1[157:186]	AJ005646.1[1903:1924:r], AM076206.1[195:212:r]
121.	***sdrM***	*tet*Efflux	-	transport-/efflux protein	hp_tetEfflux_611	AB078343.1[1808:1834]	AB078343.1[1849:1870:r]
122.	***sea***	*entA*	*sea*	enterotoxin A	entA_3,2, entA_3,3 entA-var1_11	BA000017.4 [2088572:2088598:r] BA000017.4 [2088482:2088456], BA000017.4 [2088512:2088536:r]	BA000017.4[2088473:2088492]
			sea-320E (entA-320E)	enterotoxin A, allele from strain 320E	entA-var2_11	AY196686.1|[508:532]	
		entP, sep	*sea-N315 (entP, sep)*	enterotoxin A, allele from strain N315 =enterotoxin P	entA-var3_11	BA000018.3[2011518:2011545:r]	BA000018.3[2011492:2011510]
123.	***seb***	*entB*	-	enterotoxin B	entB_11 entB_4,1	CP000046.1 [916903:916927] CP000046.1 [916583:916609]	CP000046.1[916957:916976:r] CP000046.1[916642:916663:r]
124.	***sec***	*entC*	-	enterotoxin C	entC_5,2 entC_5,3	BA000017.4 [2134733:2134761:r] BA000017.4 [2134652:2134680:r]	BA000017.4[2134593:2134610:r]
125.	***sed***	*entD*	-	enterotoxin D	entD_11	AY518388.1[49:76]	AY518388.1[79:97:r]
126.	***see***	*entE*	-	enterotoxin E	entE_11	M21319.1[601:624]	M21319.1[644:661:r]
127.	***seg***	*entG*	-	enterotoxin G	entG_11	BA000017.4 [1954500:1954526:r]	BA000017.4[1954468:1954486]
128.	***seh***	*entH*	-	enterotoxin H	entH_11	AB060536.1 [139:164]	AB060536.1[178:196:r]
129.	***sei***	*entI*	-	enterotoxin I	entG_11	BA000017.4[1957319:1957343:r]	BA000017.4[1957273:1957293]
130.	***sej***	*entJ*	-	enterotoxin J	entJ_11	AB075606.1 [1849:1876:r]	AB075606.1[1804:1823]

Nr.	Symbol	Synonymes	Alleles	Name / description of gene or gene product	probe	Probe sequence	Primer sequence
131.	***sek***	*entK*	-	enterotoxin K	hp_entK_612, hp_entK_611	AF410775.1[14220:14250], AF410775.1[13901:13932]	AF410775.1[14306:14328:r], AF410775.1[14268:14290:r], AF410775.1[13951:13974:r]
132.	***sel***	*entL*	-	enterotoxin L	entL_11	AF217235.1[892:919]	AF217235.1[930:950:r]
133.	***sem***	*entM*	-	enterotoxin M	entM_11	BA000017.4[1958262:1958291:r]	BA000017.4[1958242:1958260]]
134.	***sen***	*entN*	*sen- other than RF122*	enterotoxin N	entN_11	BA000017.4[1955741:1955768:r]	BA000017.4[1955492:1955513]
			sen-consensus		hp_entN_611	AF156894.1[1203:1230]	AF156894.1[1458:1479:r]
135.	***seo***	*entO*	-	enterotoxin O	entO_11	BA000017.4[1958936:1958962:r]	BA000017.4[1958904:1958925]
136.	***seq***	*entQ*	-	enterotoxin Q	hp_entQ_611, hp_entQ_612	AF410775.1[13213:13242], AF410775.1[13437:13467]	AF410775.1[13267:13288:r], AF410775.1[13404:13423:r], AF410775.1[13551:13570:r]
137.	***ser***	*entR*	-	enterotoxin R	entR_11	AB075606.1 [750:775]	AB075606.1[783:802:r]
138.	***"setB1"***	-	*setB1*	staphylococcal exotoxin-like protein, second locus	setB-SA1178_11	CP000046.1 [1182940:1182967:r]	CP000046.1[1182916:1182934]
			setB1-MRSA252		setB-SAR1139_11	BX571856.1 [1185070:1185094:r]	BX571856.1[1185043:1185061]
139.	***"setB2"***	-	*setB2*	staphylococcal exotoxin-like protein, second locus	setB-SA1179_11	CP000046.1 [1183750:1183777:r]	CP000046.1[1183717:1183736]
			setB2-MRSA252		setB-SAR1140_11	BX571856.1 [1185785:1185812:r]	BX571856.1[1185730:1185749]
140.	***"setB3"***	-	*setB3*	staphylococcal exotoxin-like protein, second locus	setB-SA1180_11	CP000046.1 [1184568:1184596:r]	CP000046.1[1184540:1184558]
141.	***"setC"***	*selX*	-	staphylococcal enterotoxin-like toxin X	setC-MW0345_11	CP000046.1 [446394:446420]	CP000046.1[446441:446462:r]
142.	***seu / sey***		-	Enterotoxin U and/or Y	hp_entU_611	AF156894.1[263:292],	AF156894.1[318:338:r]
143.	***spa***	-	-	Protein A	proteinA_12	CP000046.1 [107407:107435:r]	CP000046.1[107900:107917] CP000046.1[107378:107395]
144.	***splA***	-	-	serin protease A	splA_11	CP000046.1 [1921021:1921046:r]	CP000046.1[1920987:1921005]
145.	***splB***	-	-	serin protease B	splB_11	CP000046.1 [1920101:1920126:r]	CP000046.1[1920073:1920090]
146.	***splE***	-	-	serin protease E	hp_splE_611	AF271715.1[4880:4903]	AF271715.1[4921:4941:r]
147.	***ssl01***	*set6, set16*	*ssl01-COL*<br><br>*ssl01-Mu50*<br><br>*ssl01-MW2*<br><br>*ssl01-MRSA252*	staphylococcal superantigen-like protein 1**:**<br><br>set6-COL (SACOL468): probe 1_11+probe 1_12<br><br>set6-Mu50 (SAV0422): probe 1_11+probe 4_11<br><br>set6-MW2 (MW0382): probe 2_11+probe 2_12<br><br>SAR0422 (from strain MRSA 252): probe 2_11+probe 1_12	hp_set6_probe 1_11	BA000017.4[467156:467183], BA000018.3[441398:441425], AC069311.6[16791:16818]	BA000017.4[467190:467210:r], BA000018.3[441432:441452:r], AC069311.6[16825:16845:r]
					hp_set6_probe 1_12	BA000017.4[467359:467382], BA000018.3[441601:441624], BX571856.1[453059:453082]	BA000017.4[467396:467414:r], BA000018.3[441638:441656:r], BA000033.2[429765:429783:r]
					hp_set6_probe 2_11	BA000033.2[429525:429550], BX571857.1[428186:428211], BX571856.1[452856:452881]	BA000033.2[429559:429578:r], BX571857.1[428220:428239:r], BX571856.1[452890:452909:r]
					hp_set6_probe 2_12	BA000033.2[429731:429757], BX571857.1[428392:428418]	
					hp_set6_probe 4_11	AC069311.6[16994:17017], CP000046.1[470856:470879]	
			ssl01-RF122	staphylococcal superantigen-like protein 1 allele from strain RF122 (ST151)	hp_ssl01_611	AJ938182.1[412226:412252], AJ938182.1[412017:412045]	AC069311.6[17031:17049:r], AJ938182.1[412058:412076:r]
148.	***ssl02***	*set7, set17*	*ssl02*	staphylococcal superantigen-like	set7-var1_11	CP000046.1 [471582:471608]	CP000046.1[471646:471665:r]

Nr.	Symbol	Synonymes	Alleles	Name / description of gene or gene product	probe	Probe sequence	Primer sequence
			ssl02-MRSA252	protein 2	set7-var2_11	BX571856.1 [453788:453814]	CP000046.1[471646:471665:r]
149.	***ssl03***	*set8, set18*	*ssl03*	staphylococcal superantigen-like protein 3	set8_11<br>hp_ssl03_611	CP000046.1 [472456:472481]<br>AJ938182.1[413800:413827]	CP000046.1[472502:472520:r]
			ssl03-MRSA252		set-SAR0424_11	BX571856.1 [454824:454850]	
150.	***ssl04***	*set9, set19*	*ssl04-COL*	staphylococcal superantigen-like protein 4	set9-var1_11<br>set9-var1_12	CP000046.1 [474254:474283]<br>CP000046.1 [474511:474537]	CP000046.1[474328:474349:r]<br>CP000046.1[474556:474575:r]
			ssl04-MRSA252		set-SAR0425_11<br>set-SAR0425_12	BX571856.1 [456040:456064]<br>BX571856.1 [455386:455412]	BX571856.1[454869:454886:r]<br>BX571856.1[456703:456721:r]
151.	***ssl05***	*set3, set20*	*ssl05*	staphylococcal superantigen-like protein 5	set3-var1_11<br>hp_ssl05_612	BA000017.4 [470660:470688]<br>BA000017.4 [470300:470327]	BA000017.4[470703:470720:r]<br>BA000017.4[470338:470359:r]
			ssl05-MRSA252		set3-var2_11	BX571856.1 [457227:457253]	BX571856.1[457263:457284:r]
			ssl05-RF122		hp_ssl05_611	AJ938182.1[415143:415170]	AJ938182.1[415181:415202:r]
152.	***ssl06***	*set21*	*ssl06-MW2*	staphylococcal superantigen-like protein 6	set21_11<br>hp_ssl06_611	BA000033.2 [435210:435237]<br>BX571857.1 [433755:433784]	BA000033.2[435244:435264:r]<br>BA000033.2[435132:435153:r]<br>CP000253.1[394288:394308:r]
153.	***ssl07***	*set1, set22*	*ssl07*	staphylococcal superantigen-like protein 7	set1-var4_11	BA000017.4 [471560:471589]	BA000017.4[471600:471617:r]
			ssl07-FRI326		set1-var2_11	AF188836.1 [165:194]	BA000017.4[471600:471617:r]
			ssl07-MRSA252		set1-var1_11	BX571856.1 [458486:458515]	BA000017.4[471600:471617:r]
154.	***ssl08***	*set12, set23*	*ssl08*	staphylococcal superantigen-like protein 8	set12_11<br>hp_ssl08_611	BA000017.4 [472594:472621]<br>AJ938182.1 [417435:417464]	BA000017.4[472624:472644:r]<br>AJ938182.1[417465:417488:r]
155.	***ssl09***	*set5, set24*	*ssl09*	staphylococcal superantigen-like protein 9	set5-var1_11<br>hp_ssl09_611	CP000046.1 [475126:475152]<br>CP000046.1 [475125:475151]	CP000046.1[475171:475191:r]
			ssl09-MRSA252		set5-var2_11	BX571856.1 [459446:459472]	BX571856.1[459498:459519:r]
156.	***ssl10***	*set4, set25*	*ssl10*	staphylococcal superantigen-like protein 10	set4-var1_11	CP000046.1 [476429:476457]	CP000046.1[476474:476491:r]
			ssl10-RF122		hp_ssl10_611	AJ938182.1 [419736:419765]	
			ssl10-MRSA252		set4-var2_11	BX571856.1 [460746:460772]	BX571856.1[460783:460800:r]
157.	***ssl11***	*set2, set26*	*ssl11-COL*	staphylococcal superantigen-like protein 11	set2-var4_11	CP000046.1 [480340:480368]	CP000046.1[480377:480395:r]
			ssl11-MRSA252		set2-var3_11	BA000017.4 [478916:478945]	BA000017.4[478947:478967:r]
			ssl11-MW2		set2-var1_11	BA000033.2 [443788:443816]	BA000033.2[443857:443879:r]
			ssl11-Mu50		set2-var2_11	BX571856.1 [464936:464964]	BX571856.1[464979:464999:r]
158.	***sspA***	-	*sspA-C66*	glutamylendopeptidase	hp_sspA_613,<br>hp_sspA_611	AF309515.1[1298:1323],<br>AF309515.1[591:618]	AF309515.1[632:650:r],<br>AF309515.1[1327:1347:r]
			sspA-MRSA252		hp_sspA_613,<br>hp_sspA_612,<br>hp_sspA_611	AF309515.1[1298:1323],<br>BX571856.1[1064056:1064082:r],<br>AF309515.1[591:618]	AF309515.1[632:650:r],<br>AF309515.1[1327:1347:r]
			sspA-RF122		hp_sspA_611,<br>hp_sspA_614	AF309515.1[591:618],<br>AJ938182.1[991112:991137:r]	AF309515.1[632:650:r],<br>AF309515.1[1327:1347:r]
159.	***sspB***	-	-	staphopain B, protease	hp_sspB_611,<br>hp_sspB_612	AF309515.1[1546:1571],<br>AF309515.1[2231:2260]	AF309515.1[1583:1603:r],<br>AF309515.1[2264:2282:r]
160.	***sspP***	-	-	staphopain A (staphylopain A), protease	hp_sspP_612,<br>hp_sspP_611	AC090969.3[16450:16478],<br>AC090969.3[15944:15970]	AC090969.3[16483:16504:r],<br>AC090969.3[15980:15998:r]
161.	***tet*(K)**	*tetK*	-	tetracycline resistance	hp_tetK-59251_PM1,<br>hp_tetK-59250_PM1	M16217.1 [1424:1452]<br>M16217.1 [1507:1535]	M16217.1[1582:1604:r]
162.	***tet*(M)**	*tetM*	-	tetracycline resistance	tetM_11,3	BA000017.4 [440374:440400:r]	BA000017.4[440340:440358]

Nr.	Symbol	Synonymes	Alleles	Name / description of gene or gene product	probe	Probe sequence	Primer sequence
163.	***tst1***	-	*tst1_other than RF122*	toxic shock syndrome toxin 1	tst1_16,2_PM4	BA000017.4 [2137855:2137879]	BA000017.4[2137910:2137930:r]
			tst1_consensus		hp_tst_611	BA000017.4 [2138016:2138044]	BA000017.4[2138051:2138070:r]
164.	***ugpQ***	-	-	glycerophosphoryl diester phosphodiesterase, associated with *mecA*	hp_ugpQ_611	AB033763.2[34349:34374:r]	AB033763.2[34320:34338]
165.	***vanA***	-	-	vancomycin resistance gene	18,2-vanA	AB247327.1[16135:16162]	AB247327.1[16238:16257:r]
166.	***vanB***	-	-	vancomycin resistance gene from enterococci and *Clostridium*	19,3-vanB<br>vanB_11	AE016830.1[2213146:2213171:r],<br>AE016830.1[2213149:2213173:r]	AE016830.1[2213051:2213068]
167.	***vanZ***	-	-	teicoplanin resistance gene from enterococci	20,3-vanZ	AB247327.1[19318:19343]	AB247327.1[19367:19386:r]
168.	***vatA***	-	-	virginiamycin A acetyltransferase	vatA_15,3	AF117258.1[2296:2323:r]	AF117258.1[2229:2247]
169.	***vatB***	-	-	acetyltransferase inactivating streptogramin A	vatB_16,3	U19459.1[543:570]	U19459.1[659:680:r]
170.	***vga***	-	*vga*	ATP binding protein, streptogramin-A-resistance	vga_17,3	AF117259.1[3729:3756]	AF117259.1[3925:3944:r],<br>AF117259.1[3779:3799:r]
171.			*vga-BM 3327*		vgaA_18,3	AF186237.2[6470:6497]	AF186237.2[6559:6578:r]
172.	***vgb***	-	-	virginiamycin B hydrolase	vgb_19,2	AF117258.1[3342:3369:r]	AF117258.1[3261:3283]
173.	***vraS***	-	-	sensor protein	hp_vraS_612	AB035448.1[4748:4773]	AB035448.1[4783:4802:r]
174.	***vwb***	-	*vwb (cons)*	van Willebrand factor binding protein	hp_vwb_615	CP000046.1 [884859:884884]	CP000046.1[883877:883899:r]
		-	*vwb (COL+MW2)*		hp_vwb_612	CP000046.1 [883836:883864]	CP000046.1[884895:884914:r]
			vwb (MRSA252)		hp_vwb_613	BX571856.1 [891899:891927]	BX571856.1[891952:891972:r]
		-	*vwb (Mu50)*		hp_vwb_614	BA000017.4 [890919:890947]	BA000017.4[890960:890980:r]<br>BA000017.4[891393:891412:r]
		-	*vwb (RF122)*		hp_vwb_611	AJ938182.1 [821019:821046]	AJ938182.1[821064:821083:r]
175.	***xylR***	*mecR2*	-	homolog of xylose repressor, associated with SCC*mec*-elements	hp_xylR_611	AB037671.1[21440:21468:r]	AB037671.1[21410:21429]
