# Supplementary material for: Characterisation of a Staphylococcus aureus Isolate Carrying Phage-Borne Enterotoxin E from a European Badger (Meles meles)
Source: Pathogens. 2023 May 12;12(5):704. doi: 10.3390/pathogens12050704 (PMC10220527; doi:10.3390/pathogens12050704)
Supplement: Supplementary file 1 [file pathogens-12-00704-s001.zip › Supplemental File 2 Full Hybridisation profiles.pdf]

STRAIN / ISOLATE	ACESSION NUMBER	SPECIES MARKER							STAPHYLOXANTHIN BIOSYNTHESIS OPERON					REGULATORY GENES		
		rrnD1	gapA	katA	CoA	nuc1	spa	sbi	crtM		crtN	crtO	crtP	sarA	saeS	vraS
									crtM-nonST93	crtM-ST93						
		Domain 1 of 23S-rRNA	glyceraldehyde 3-phosphate dehydrogenase, locus 1	katalase A	coagulase	thermostable extracellular nuclease	staphylococcal protein A	IgG-binding protein	dehydrosqualene synthase		dehydrosqualene desaturase	staphyloxanthin acyltransferase	diaponeuro-sporene oxidase	staphylococcal accessory regulator A	histidine protein kinase, sae locus	sensor protein
V40-124624	[pending]	POS	POS	POS	POS	POS	POS	POS	POS	NEG	POS	POS	POS	POS	POS	POS
V40-124624: in silico prediction	[pending]	POS	POS	POS	POS	POS	POS	POS	POS	NEG	POS	POS	POS	POS	POS	POS
SA-120: in silico prediction	GenBank JXIG/SAMN03219992	POS	POS	POS	POS	POS	POS	POS	POS	NEG	POS	POS	POS	POS	POS	POS
61908: in silico prediction	GenBank FJNO/SAMEA2298602	POS	POS	POS	POS	POS	POS	POS	POS	NEG	POS	POS	POS	POS	POS	POS
M1790_98_1: in silico prediction	GenBank FJNU/SAMEA1929516	POS	POS	POS	POS	POS	POS	POS	POS	NEG	POS	POS	POS	POS	POS	POS
GKP138-13: in silico prediction	GenBank FMRL/SAMEA1708807	POS	POS	POS	POS	POS	POS	POS	POS	NEG	POS	POS	POS	POS	POS	POS
GKP138-2: in silico prediction	GenBank FMRM/SAMEA1708877	POS	POS	POS	POS	POS	POS	POS	POS	NEG	POS	POS	POS	POS	POS	POS
GKP138-31: in silico prediction	GenBank FMRN/SAMEA1708981	POS	POS	POS	POS	POS	POS	POS	POS	NEG	POS	POS	POS	POS	POS	POS
GKP138-33: in silico prediction	GenBank FMRO/SAMEA1708977	POS	POS	POS	POS	POS	POS	POS	POS	NEG	POS	POS	POS	POS	POS	POS
GKP138-52: in silico prediction	GenBank FMRP/SAMEA1708955	POS	POS	POS	POS	POS	POS	POS	POS	NEG	POS	POS	POS	POS	POS	POS
GKP138-78: in silico prediction	GenBank FMRR/SAMEA1708688	POS	POS	POS	POS	POS	POS	POS	POS	NEG	POS	POS	POS	POS	POS	POS
GKP136-4: in silico prediction	GenBank FMRV/SAMEA1708785	POS	POS	POS	POS	POS	POS	POS	POS	NEG	POS	POS	POS	POS	POS	POS
ZTA09_03668_9_HSA: in silico prediction	SAMEA2298561	POS	POS	POS	POS	POS	POS	POS	POS	NEG	POS	POS	POS	POS	POS	POS
GKP136-58: in silico prediction	GenBank FMRJ/SAMEA1708766	POS	POS	POS	POS	POS	POS	POS	POS	NEG	POS	POS	POS	POS	POS	POS
004_0004_23k: in silico prediction	SAMEA2298547	POS	POS	POS	POS	POS	POS	POS	POS	NEG	POS	POS	POS	POS	POS	POS
BA06_02038: in silico prediction	SAMEA2298548	POS	POS	POS	POS	POS	POS	POS	POS	NEG	POS	POS	POS	POS	POS	POS
GKP138-4: in silico prediction	GenBank FMRQ/SAMEA1708883	POS	POS	POS	POS	POS	POS	POS	POS	NEG	POS	POS	POS	POS	POS	POS
ZTA10_02421_9_HSA: in silico prediction	SAMEA2298571	POS	POS	POS	POS	POS	POS	POS	POS	NEG	POS	POS	POS	POS	POS	POS
GKP136-62: in silico prediction	GenBank FMSC/SAMEA1708769	POS	POS	POS	POS	POS	POS	POS	POS	NEG	POS	POS	POS	POS	POS	POS
LGA251: in silico prediction	GenBank FR821779/SAMEA2272771	POS	POS	POS	POS	POS	POS	POS	POS	NEG	POS	POS	POS	POS	POS	POS
UK_NCTC13552: in silico prediction	GenBank LR134084/SAMEA3491693	POS	POS	POS	POS	POS	POS	POS	POS	NEG	POS	POS	POS	POS	POS	POS
GKP138-71: in silico prediction	GenBank FMPY/SAMEA1708930	POS	POS	POS	POS	POS	POS	POS	POS	NEG	POS	POS	POS	POS	POS	POS
cat-mandible-2013: in silico prediction	GenBank LUFG/SAMN04537353	POS	POS	POS	POS	POS	POS	POS	POS	NEG	POS	POS	POS	POS	POS	POS
10_7550_D: in silico prediction	SAMEA1033305	POS	POS	POS	POS	POS	POS	POS	POS	NEG	POS	POS	POS	POS	POS	POS
H114440275_A: in silico prediction	SAMEA1904132	POS	POS	POS	POS	POS	POS	POS	POS	NEG	POS	POS	POS	POS	POS	POS

STRAIN / ISOLATE	REGULATORY GENES													
												agrV (argenteus)		hld
	agrB-I	agrC-I	agrD-I	agrB-II	agrC-II	agrD-II	agrB-III	agrC-III	agrD-III	agrB-IV	agrC-IV	agrV-ST1850	agrV-ST2198	
												ory gene regulator alleles from S. argenteus		haemolysin delta
**V40-124624**	NEG	NEG	NEG	POS	POS	POS	NEG	NEG	NEG	NEG	NEG	NEG	NEG	POS
**V40-124624: in silico prediction**	NEG	NEG	NEG	POS	POS	POS	NEG	NEG	NEG	NEG	NEG	NEG	NEG	POS
**SA-120: in silico prediction**	NEG	NEG	NEG	POS	POS	POS	NEG	NEG	NEG	NEG	NEG	NEG	NEG	POS
**61908: in silico prediction**	NEG	NEG	NEG	POS	POS	POS	NEG	NEG	NEG	NEG	NEG	NEG	NEG	POS
**M1790_98_1: in silico prediction**	NEG	NEG	NEG	POS	POS	POS	NEG	NEG	NEG	NEG	NEG	NEG	NEG	POS
**GKP138-13: in silico prediction**	NEG	NEG	NEG	POS	POS	POS	NEG	NEG	NEG	NEG	NEG	NEG	NEG	POS
**GKP138-2: in silico prediction**	NEG	NEG	NEG	POS	POS	POS	NEG	NEG	NEG	NEG	NEG	NEG	NEG	POS
**GKP138-31: in silico prediction**	NEG	NEG	NEG	POS	POS	POS	NEG	NEG	NEG	NEG	NEG	NEG	NEG	POS
**GKP138-33: in silico prediction**	NEG	NEG	NEG	POS	POS	POS	NEG	NEG	NEG	NEG	NEG	NEG	NEG	POS
**GKP138-52: in silico prediction**	NEG	NEG	NEG	POS	POS	POS	NEG	NEG	NEG	NEG	NEG	NEG	NEG	POS
**GKP138-78: in silico prediction**	NEG	NEG	NEG	POS	POS	POS	NEG	NEG	NEG	NEG	NEG	NEG	NEG	POS
**GKP136-4: in silico prediction**	NEG	NEG	NEG	POS	POS	POS	NEG	NEG	NEG	NEG	NEG	NEG	NEG	POS
**ZTA09_03668_9_HSA: in silico prediction**	NEG	NEG	NEG	POS	POS	POS	NEG	NEG	NEG	NEG	NEG	NEG	NEG	POS
**GKP136-58: in silico prediction**	NEG	NEG	NEG	POS	POS	POS	NEG	NEG	NEG	NEG	NEG	NEG	NEG	POS
**004_0004_23k: in silico prediction**	NEG	NEG	NEG	POS	POS	POS	NEG	NEG	NEG	NEG	NEG	NEG	NEG	POS
**BA06_02038: in silico prediction**	NEG	NEG	NEG	POS	POS	POS	NEG	NEG	NEG	NEG	NEG	NEG	NEG	POS
**GKP138-4: in silico prediction**	NEG	NEG	NEG	POS	POS	POS	NEG	NEG	NEG	NEG	NEG	NEG	NEG	POS
**ZTA10_02421_9_HSA: in silico prediction**	NEG	NEG	NEG	POS	POS	POS	NEG	NEG	NEG	NEG	NEG	NEG	NEG	POS
**GKP136-62: in silico prediction**	NEG	NEG	NEG	POS	POS	POS	NEG	NEG	NEG	NEG	NEG	NEG	NEG	POS
**LGA251: in silico prediction**	NEG	NEG	NEG	POS	POS	POS	NEG	NEG	NEG	NEG	NEG	NEG	NEG	POS
**UK_NCTC13552: in silico prediction**	NEG	NEG	NEG	POS	POS	POS	NEG	NEG	NEG	NEG	NEG	NEG	NEG	POS
**GKP138-71: in silico prediction**	NEG	NEG	NEG	POS	POS	POS	NEG	NEG	NEG	NEG	NEG	NEG	NEG	POS
**cat-mandible-2013: in silico prediction**	NEG	NEG	NEG	POS	POS	POS	NEG	NEG	NEG	NEG	NEG	NEG	NEG	POS
**10_7550_D: in silico prediction**	NEG	NEG	NEG	POS	POS	POS	NEG	NEG	NEG	NEG	NEG	NEG	NEG	POS
**H114440275_A: in silico prediction**	NEG	NEG	NEG	POS	POS	POS	NEG	NEG	NEG	NEG	NEG	NEG	NEG	POS

STRAIN / ISOLATE	METHICILLIN RESISTANCE AND SCCmec TYPING									
	ugpQ	mecA	delta_mecR1	mecR1	mecI	fudoh-PSM	cstB-SCC1 (ex Q2G1R6) (SCCmec II/III)	xylR/mecR2	mecC	blaZ (SCCmec XI)
	Glycerophosphoryl diester phosphodiesterase. Accompanies mecA in nearly all SCCmec sequences.	Modified penicillin binding protein (PBP2a)	Truncated methicillin resistance operon repressor 1. Truncated mecR1 is present in SCCmec I, IV, V, VI, VII; complete absence of mecR1 from SCCmec V, IX, X	Methicillin resistance operon repressor 1. Un-truncated sequence in SCCmec II, SCCmec III, SCCmec VIII	Methicillin-resistance regulatory protein. Present in SCCmec II (although absent from Irish SCCmec II variants C and E), SCCmec III, SCCmec VIII	Phenol soluble modulin from SCCmec	CsoR-like sulfur transferase-regulated genes B/metallo-beta-lactamase superfamily protein.	Methicillin resistance operon repressor 2, Homolog of xylose repressor. Located next to mec operon downstream of mecI (not present if mecI is truncated)	Alternate gene encoding a modified penicillin binding protein . Present in, and characteristic for, SCCmec XI	Beta-lactamase from SCCmec XI
V40-124624	NEG	NEG	NEG	NEG	NEG	NEG	NEG	NEG	neg	NEG
V40-124624: in silico prediction	NEG	NEG	NEG	NEG	NEG	NEG	NEG	NEG	neg	NEG
SA-120: in silico prediction	NEG	NEG	NEG	NEG	NEG	NEG	NEG	NEG	NEG	NEG
61908: in silico prediction	NEG	NEG	NEG	NEG	NEG	NEG	NEG	NEG	NEG	NEG
M1790_98_1: in silico prediction	NEG	NEG	NEG	NEG	NEG	NEG	NEG	NEG	NEG	NEG
GKP138-13: in silico prediction	NEG	NEG	NEG	NEG	NEG	NEG	NEG	NEG	NEG	NEG
GKP138-2: in silico prediction	NEG	NEG	NEG	NEG	NEG	NEG	NEG	NEG	NEG	NEG
GKP138-31: in silico prediction	NEG	NEG	NEG	NEG	NEG	NEG	NEG	NEG	NEG	NEG
GKP138-33: in silico prediction	NEG	NEG	NEG	NEG	NEG	NEG	NEG	NEG	NEG	NEG
GKP138-52: in silico prediction	NEG	NEG	NEG	NEG	NEG	NEG	NEG	NEG	NEG	NEG
GKP138-78: in silico prediction	NEG	NEG	NEG	NEG	NEG	NEG	NEG	NEG	NEG	NEG
GKP136-4: in silico prediction	NEG	NEG	NEG	NEG	NEG	NEG	NEG	NEG	NEG	NEG
ZTA09_03668_9_HSA: in silico prediction	NEG	NEG	NEG	NEG	NEG	NEG	NEG	NEG	NEG	NEG
GKP136-58: in silico prediction	NEG	NEG	NEG	NEG	NEG	NEG	NEG	NEG	NEG	NEG
004_0004_23k: in silico prediction	NEG	NEG	NEG	NEG	NEG	NEG	NEG	NEG	NEG	NEG
BA06_02038: in silico prediction	NEG	NEG	NEG	NEG	NEG	NEG	NEG	NEG	NEG	NEG
GKP138-4: in silico prediction	NEG	NEG	NEG	NEG	NEG	NEG	NEG	NEG	NEG	NEG
ZTA10_02421_9_HSA: in silico prediction	NEG	NEG	NEG	NEG	NEG	NEG	NEG	NEG	NEG	NEG
GKP136-62: in silico prediction	NEG	NEG	NEG	NEG	NEG	NEG	NEG	NEG	NEG	NEG
LGA251: in silico prediction	NEG	NEG	NEG	NEG	NEG	NEG	NEG	NEG	POS	POS
UK_NCTC13552: in silico prediction	NEG	NEG	NEG	NEG	NEG	NEG	NEG	NEG	POS	POS
GKP138-71: in silico prediction	NEG	NEG	NEG	NEG	NEG	NEG	NEG	NEG	POS	POS
cat-mandible-2013: in silico prediction	NEG	NEG	NEG	NEG	NEG	NEG	NEG	NEG	POS	POS
10_7550_D: in silico prediction	NEG	NEG	NEG	NEG	NEG	NEG	NEG	NEG	POS	POS
H114440275_A: in silico prediction	NEG	NEG	NEG	NEG	NEG	NEG	NEG	NEG	POS	POS

STRAIN / ISOLATE	METHICILLIN RESISTANCE AND SCCmec TYPING																		
	plsSCC (COL)	mvaS-SCC	Q5HJW6	Q7A207	cstB-SCC2 (Q2G1R6)	Q9S0M4	kdpA-SCC	kdpB-SCC	kdpC-SCC	kdpD-SCC	kdpE-SCC	Q9XB68-dcs	Q93IB7	D1GU38		Q933A2	D1GU55	F9JXC0	
														D1GU38	D1GU38 (TW20)			F9JXC0 (full)	F9JXC0 (trunc)
	Plasmin-sensitive surface protein, prevents bacterial adhesion in vitro, located in SCC, close to mec operon. Subtyping SCCmec I	Truncated 3-hydroxy-3-methylglutaryl CoA synthase. Subtyping SCCmec I, II, IV, V	Putative protein next to dru	Putative protein	CsoR-like sulfur transferase-regulated genes B/metallo-beta-lactamase superfamily protein.	Putative protein. Subtyping SCCmec I, SCCmec/ACME composites and SCCmec from WA40	Potassium-translocating ATPase A, chain 2	Potassium-transporting ATPase B, chain 1	Potassium-translocating ATPase C, chain 2	Sensor kinase protein	KDP operon transcriptional regulatory protein	Located at the terminus of SCCmec next to orfX.This locus comprises the downstream constant segment (dcs) that in turn comprises a copy of the SCC direct repeat	LytTR domain DNA-binding regulator. Subtyping SCCmec III	Subtyping SCCmec III, identification of SCCmec VT, SCCmec ZH47, SCCmec VII because of an association with (additional/second) ccrC copies	Putative protein	Putative ADP-ribosyltransferase. Subtyping SCCmec III and SCCmec IX	Putative membrane protein. Subtyping SCCmec III, additional marker for SCCmec VII	KDP operon transcriptional regulatory protein	
V40-124624	NEG	NEG	NEG	NEG	NEG	NEG	NEG	NEG	NEG	NEG	NEG	NEG	NEG	NEG	NEG	NEG	NEG	NEG	NEG
V40-124624: in silico prediction	NEG	NEG	NEG	NEG	NEG	NEG	NEG	NEG	NEG	NEG	NEG	NEG	NEG	NEG	NEG	NEG	NEG	NEG	NEG
SA-120: in silico prediction	NEG	NEG	NEG	NEG	NEG	NEG	NEG	NEG	NEG	NEG	NEG	NEG	NEG	NEG	NEG	NEG	NEG	NEG	NEG
61908: in silico prediction	NEG	NEG	NEG	NEG	NEG	NEG	NEG	NEG	NEG	NEG	NEG	NEG	NEG	NEG	NEG	NEG	NEG	NEG	NEG
M1790_98_1: in silico prediction	NEG	NEG	NEG	NEG	NEG	NEG	NEG	NEG	NEG	NEG	NEG	NEG	NEG	NEG	NEG	NEG	NEG	NEG	NEG
GKP138-13: in silico prediction	NEG	NEG	NEG	NEG	NEG	NEG	NEG	NEG	NEG	NEG	NEG	NEG	NEG	NEG	NEG	NEG	NEG	NEG	NEG
GKP138-2: in silico prediction	NEG	NEG	NEG	NEG	NEG	NEG	NEG	NEG	NEG	NEG	NEG	NEG	NEG	NEG	NEG	NEG	NEG	NEG	NEG
GKP138-31: in silico prediction	NEG	NEG	NEG	NEG	NEG	NEG	NEG	NEG	NEG	NEG	NEG	NEG	NEG	NEG	NEG	NEG	NEG	NEG	NEG
GKP138-33: in silico prediction	NEG	NEG	NEG	NEG	NEG	NEG	NEG	NEG	NEG	NEG	NEG	NEG	NEG	NEG	NEG	NEG	NEG	NEG	NEG
GKP138-52: in silico prediction	NEG	NEG	NEG	NEG	NEG	NEG	NEG	NEG	NEG	NEG	NEG	NEG	NEG	NEG	NEG	NEG	NEG	NEG	NEG
GKP138-78: in silico prediction	NEG	NEG	NEG	NEG	NEG	NEG	NEG	NEG	NEG	NEG	NEG	NEG	NEG	NEG	NEG	NEG	NEG	NEG	NEG
GKP136-4: in silico prediction	NEG	NEG	NEG	NEG	NEG	NEG	NEG	NEG	NEG	NEG	NEG	NEG	NEG	NEG	NEG	NEG	NEG	NEG	NEG
ZTA09_03668_9_HSA: in silico prediction	NEG	NEG	NEG	NEG	NEG	NEG	NEG	NEG	NEG	NEG	NEG	NEG	NEG	NEG	NEG	NEG	NEG	NEG	NEG
GKP136-58: in silico prediction	NEG	NEG	NEG	NEG	NEG	NEG	NEG	NEG	NEG	NEG	NEG	NEG	NEG	NEG	NEG	NEG	NEG	NEG	NEG
004_0004_23k: in silico prediction	NEG	NEG	NEG	NEG	NEG	NEG	NEG	NEG	NEG	NEG	NEG	NEG	NEG	NEG	NEG	NEG	NEG	NEG	NEG
BA06_02038: in silico prediction	NEG	NEG	NEG	NEG	NEG	NEG	NEG	NEG	NEG	NEG	NEG	NEG	NEG	NEG	NEG	NEG	NEG	NEG	NEG
GKP138-4: in silico prediction	NEG	NEG	NEG	NEG	NEG	NEG	NEG	NEG	NEG	NEG	NEG	NEG	NEG	NEG	NEG	NEG	NEG	NEG	NEG
ZTA10_02421_9_HSA: in silico prediction	NEG	NEG	NEG	NEG	NEG	NEG	NEG	NEG	NEG	NEG	NEG	NEG	NEG	NEG	NEG	NEG	NEG	NEG	NEG
GKP136-62: in silico prediction	NEG	NEG	NEG	POS	NEG	NEG	NEG	NEG	NEG	NEG	NEG	NEG	NEG	NEG	NEG	NEG	NEG	NEG	NEG
LGA251: in silico prediction	NEG	NEG	NEG	NEG	NEG	NEG	NEG	NEG	NEG	NEG	NEG	NEG	NEG	NEG	NEG	NEG	NEG	NEG	NEG
UK_NCTC13552: in silico prediction	NEG	NEG	NEG	NEG	NEG	NEG	NEG	NEG	NEG	NEG	NEG	NEG	NEG	NEG	NEG	NEG	NEG	NEG	NEG
GKP138-71: in silico prediction	NEG	NEG	NEG	NEG	NEG	NEG	NEG	NEG	NEG	NEG	NEG	NEG	NEG	NEG	NEG	NEG	NEG	NEG	NEG
cat-mandible-2013: in silico prediction	NEG	NEG	NEG	NEG	NEG	NEG	NEG	NEG	NEG	NEG	NEG	NEG	NEG	NEG	NEG	NEG	NEG	NEG	NEG
10_7550_D: in silico prediction	NEG	NEG	NEG	NEG	NEG	NEG	NEG	NEG	NEG	NEG	NEG	NEG	NEG	NEG	NEG	NEG	NEG	NEG	NEG
H114440275_A: in silico prediction	NEG	NEG	NEG	NEG	NEG	NEG	NEG	NEG	NEG	NEG	NEG	NEG	NEG	NEG	NEG	NEG	NEG	NEG	NEG

STRAIN / ISOLATE	METHICILLIN RESISTANCE AND SCCmec TYPING															
							Q4LAG7					cas1				
	B2Y834	B6VQU0	Q3YK51	ydhK	C1PH94	DUF1958	Q4LAG7 (SCCmecV, SO385)	Q4LAG7 (SCCfus 45394F/MSSA 476)	C5QAP8 (SCCmec XI)	Q8CU82	D3JD07	cas1 (MSHR1132)	cas1 (M06-171)	fusC (Q6GD50)	tirS	yeeA
	Abortive phage resistance protein. Subtyping SCCmec IV, i.e., identification of SCCmec IV A, G, c and SCCmec MRSAZH47	Putative protein. Subtyping SCCmec IV, i.e., identification of SCCmec IVh/j	Putative protein. Subtyping SCCmec IV, i.e., identification of SCCmec IV g	Putative lipoprotein. Present in some composite elements comprising SCCmec and heavy metal resistance genes including the one in FPR3757, GenBank: CP000255.1	Putative protein	Putative protein. Subtyping SCCmec VT. Present, e.g., in PM1, GenBank BAFA but absent, e.g., in Strain 3957, GenBank AOFU	Putative protein located within SCCmec type V elements	Putative protein located within SCCfus elements	Putative protein. Identification of SCCmec XI	Putative protein. Present in some SCCmec/fus composite elements.	Putative protein. Present in some composite SCC elements	CRISPR-associated endonuclease 1. Present in MSHR1132	CRISPR-associated endonuclease 1. Present in M06/0171, GenBank HE980450.1	SCC-associated fusidic acid resistance gene. Present in "SCCfus" elements or together with mecA in composite elements.	Staphylococcal TIR-protein binding protein. Subtyping SCCfus because it is frequently, but not always, accompanying fusC	Putative DNA methyltransferase. Subtyping SCCmecIV/fus composite elements
V40-124624	NEG	NEG	NEG	NEG	NEG	NEG	NEG	NEG	NEG	NEG	NEG	NEG	NEG	NEG	NEG	NEG
V40-124624: in silico prediction	NEG	NEG	NEG	NEG	NEG	NEG	NEG	NEG	NEG	NEG	NEG	NEG	NEG	NEG	NEG	NEG
SA-120: in silico prediction	NEG	NEG	NEG	NEG	NEG	NEG	NEG	NEG	NEG	NEG	NEG	NEG	NEG	NEG	NEG	NEG
61908: in silico prediction	NEG	NEG	NEG	NEG	NEG	NEG	NEG	NEG	NEG	NEG	NEG	NEG	NEG	NEG	NEG	NEG
M1790_98_1: in silico prediction	NEG	NEG	NEG	NEG	NEG	NEG	NEG	NEG	NEG	NEG	NEG	NEG	NEG	NEG	NEG	NEG
GKP138-13: in silico prediction	NEG	NEG	NEG	NEG	NEG	NEG	NEG	NEG	NEG	NEG	NEG	NEG	NEG	NEG	NEG	NEG
GKP138-2: in silico prediction	NEG	NEG	NEG	NEG	NEG	NEG	NEG	NEG	NEG	NEG	NEG	NEG	NEG	NEG	NEG	NEG
GKP138-31: in silico prediction	NEG	NEG	NEG	NEG	NEG	NEG	NEG	NEG	NEG	NEG	NEG	NEG	NEG	NEG	NEG	NEG
GKP138-33: in silico prediction	NEG	NEG	NEG	NEG	NEG	NEG	NEG	NEG	NEG	NEG	NEG	NEG	NEG	NEG	NEG	NEG
GKP138-52: in silico prediction	NEG	NEG	NEG	NEG	NEG	NEG	NEG	NEG	NEG	NEG	NEG	NEG	NEG	NEG	NEG	NEG
GKP138-78: in silico prediction	NEG	NEG	NEG	NEG	NEG	NEG	NEG	NEG	NEG	NEG	NEG	NEG	NEG	NEG	NEG	NEG
GKP136-4: in silico prediction	NEG	NEG	NEG	NEG	NEG	NEG	NEG	NEG	NEG	NEG	NEG	NEG	NEG	NEG	NEG	NEG
ZTA09_03668_9_HSA: in silico prediction	NEG	NEG	NEG	NEG	NEG	NEG	NEG	NEG	NEG	NEG	NEG	NEG	NEG	NEG	NEG	NEG
GKP136-58: in silico prediction	NEG	NEG	NEG	NEG	NEG	NEG	NEG	NEG	NEG	NEG	NEG	NEG	NEG	NEG	NEG	NEG
004_0004_23k: in silico prediction	NEG	NEG	NEG	NEG	NEG	NEG	NEG	NEG	NEG	NEG	NEG	NEG	NEG	NEG	NEG	NEG
BA06_02038: in silico prediction	NEG	NEG	NEG	NEG	NEG	NEG	NEG	NEG	NEG	NEG	NEG	NEG	NEG	NEG	NEG	NEG
GKP138-4: in silico prediction	NEG	NEG	NEG	NEG	NEG	NEG	NEG	NEG	NEG	NEG	NEG	NEG	NEG	NEG	NEG	NEG
ZTA10_02421_9_HSA: in silico prediction	NEG	NEG	NEG	NEG	NEG	NEG	NEG	NEG	NEG	NEG	NEG	NEG	NEG	NEG	NEG	NEG
GKP136-62: in silico prediction	NEG	NEG	NEG	NEG	NEG	NEG	NEG	NEG	NEG	NEG	NEG	NEG	NEG	NEG	NEG	NEG
LGA251: in silico prediction	NEG	NEG	NEG	NEG	NEG	NEG	NEG	NEG	POS	NEG	NEG	NEG	NEG	NEG	NEG	NEG
UK_NCTC13552: in silico prediction	NEG	NEG	NEG	NEG	NEG	NEG	NEG	NEG	POS	NEG	NEG	NEG	NEG	NEG	NEG	NEG
GKP138-71: in silico prediction	NEG	NEG	NEG	NEG	NEG	NEG	NEG	NEG	POS	NEG	NEG	NEG	NEG	NEG	NEG	NEG
cat-mandible-2013: in silico prediction	NEG	NEG	NEG	NEG	NEG	NEG	NEG	NEG	POS	NEG	NEG	NEG	NEG	NEG	NEG	NEG
10_7550_D: in silico prediction	NEG	NEG	NEG	NEG	NEG	NEG	NEG	NEG	POS	NEG	NEG	NEG	NEG	NEG	NEG	NEG
H114440275_A: in silico prediction	NEG	NEG	NEG	NEG	NEG	NEG	NEG	NEG	POS	NEG	NEG	NEG	NEG	NEG	NEG	NEG

STRAIN / ISOLATE	METHICILLIN RESISTANCE AND SCCmec TYPING																	
	ACME								ccrA/B-01		ccrA/B-02		ccrA/B-03		ccrAA/C		ccrA/B-04	
	arcA-SCC	arcB-SCC	arcC-SCC	arcD-SCC	opp3B	opp3C	adhC	speG	ccrA-1	ccrB-1	ccrA-2	ccrB-2	ccrA-3	ccrB-3	ccrAA	ccrC (85-2082)	ccrA-4	ccrB-4
	Arginine deiminase	Ornithine carbamoyltransferase	Carbamate kinase	Arginine/ornithine antiporter	Oligopeptide permease, channel-forming protein	Oligopeptide permease, channel-forming protein	Alcohol dehydrogenase, zinc-containing	Spermidine N-acetyltransferase. Usually associated with ACME or composite SCCmec/ACME elements	Cassette chromosome recombinase A, type 1	Cassette chromosome recombinase B, type 1	Cassette chromosome recombinase A, type 2	Cassette chromosome recombinase B, type 2	Cassette chromosome recombinase A, type 3	Cassette chromosome recombinase B, type 3	hypoth. Protein associated with ccrC	Cassette chromosome recombinase C	Cassette chromosome recombinase A, type 4	Cassette chromosome recombinase B, type 4
V40-124624	NEG	NEG	NEG	NEG	NEG	NEG	NEG	NEG	NEG	NEG	NEG	NEG	NEG	NEG	NEG	NEG	NEG	NEG
V40-124624: in silico prediction	NEG	NEG	NEG	NEG	NEG	NEG	NEG	NEG	NEG	NEG	NEG	NEG	NEG	NEG	NEG	NEG	NEG	NEG
SA-120: in silico prediction	NEG	NEG	NEG	NEG	NEG	NEG	NEG	NEG	NEG	NEG	NEG	NEG	NEG	NEG	NEG	NEG	NEG	NEG
61908: in silico prediction	NEG	NEG	NEG	NEG	NEG	NEG	NEG	NEG	NEG	NEG	NEG	NEG	NEG	NEG	NEG	NEG	NEG	NEG
M1790_98_1: in silico prediction	NEG	NEG	NEG	NEG	NEG	NEG	NEG	NEG	NEG	NEG	NEG	NEG	NEG	NEG	NEG	NEG	NEG	NEG
GKP138-13: in silico prediction	NEG	NEG	NEG	NEG	NEG	NEG	NEG	NEG	NEG	NEG	NEG	NEG	NEG	NEG	NEG	NEG	NEG	NEG
GKP138-2: in silico prediction	NEG	NEG	NEG	NEG	NEG	NEG	NEG	NEG	NEG	NEG	NEG	NEG	NEG	NEG	NEG	NEG	NEG	NEG
GKP138-31: in silico prediction	NEG	NEG	NEG	NEG	NEG	NEG	NEG	NEG	NEG	NEG	NEG	NEG	NEG	NEG	NEG	NEG	NEG	NEG
GKP138-33: in silico prediction	NEG	NEG	NEG	NEG	NEG	NEG	NEG	NEG	NEG	NEG	NEG	NEG	NEG	NEG	NEG	NEG	NEG	NEG
GKP138-52: in silico prediction	NEG	NEG	NEG	NEG	NEG	NEG	NEG	NEG	NEG	NEG	NEG	NEG	NEG	NEG	NEG	NEG	NEG	NEG
GKP138-78: in silico prediction	NEG	NEG	NEG	NEG	NEG	NEG	NEG	NEG	NEG	NEG	NEG	NEG	NEG	NEG	NEG	NEG	NEG	NEG
GKP136-4: in silico prediction	NEG	NEG	NEG	NEG	NEG	NEG	NEG	NEG	NEG	NEG	NEG	NEG	NEG	NEG	NEG	NEG	NEG	NEG
ZTA09_03668_9_HSA: in silico prediction	NEG	NEG	NEG	NEG	NEG	NEG	NEG	NEG	NEG	NEG	NEG	NEG	NEG	NEG	NEG	NEG	NEG	NEG
GKP136-58: in silico prediction	NEG	NEG	NEG	NEG	NEG	NEG	NEG	NEG	NEG	NEG	NEG	NEG	NEG	NEG	NEG	NEG	NEG	NEG
004_0004_23k: in silico prediction	NEG	NEG	NEG	NEG	NEG	NEG	NEG	NEG	NEG	NEG	NEG	NEG	NEG	NEG	NEG	NEG	NEG	NEG
BA06_02038: in silico prediction	NEG	NEG	NEG	NEG	NEG	NEG	NEG	NEG	NEG	NEG	NEG	NEG	NEG	NEG	NEG	NEG	NEG	NEG
GKP138-4: in silico prediction	NEG	NEG	NEG	NEG	NEG	NEG	NEG	NEG	NEG	NEG	NEG	NEG	NEG	NEG	NEG	NEG	NEG	NEG
ZTA10_02421_9_HSA: in silico prediction	NEG	NEG	NEG	NEG	NEG	NEG	NEG	NEG	NEG	NEG	NEG	NEG	NEG	NEG	NEG	NEG	NEG	NEG
GKP136-62: in silico prediction	NEG	NEG	NEG	NEG	NEG	NEG	NEG	NEG	POS	POS	NEG	NEG	NEG	NEG	NEG	NEG	NEG	NEG
LGA251: in silico prediction	NEG	NEG	NEG	NEG	NEG	NEG	NEG	NEG	NEG	AMB	NEG	NEG	NEG	NEG	NEG	NEG	NEG	NEG
UK_NCTC13552: in silico prediction	NEG	NEG	NEG	NEG	NEG	NEG	NEG	NEG	NEG	AMB	NEG	NEG	NEG	NEG	NEG	NEG	NEG	NEG
GKP138-71: in silico prediction	NEG	NEG	NEG	NEG	NEG	NEG	NEG	NEG	NEG	AMB	NEG	NEG	NEG	NEG	NEG	NEG	NEG	NEG
cat-mandible-2013: in silico prediction	NEG	NEG	NEG	NEG	NEG	NEG	NEG	NEG	NEG	AMB	NEG	NEG	NEG	NEG	NEG	NEG	NEG	NEG
10_7550_D: in silico prediction	NEG	NEG	NEG	NEG	NEG	NEG	NEG	NEG	NEG	AMB	NEG	NEG	NEG	NEG	NEG	NEG	NEG	NEG
H114440275_A: in silico prediction	NEG	NEG	NEG	NEG	NEG	NEG	NEG	NEG	NEG	AMB	NEG	NEG	NEG	NEG	NEG	NEG	NEG	NEG

STRAIN / ISOLATE	HEAVY METAL RESISTANCES, SCCmec AND OTHERS											
	merA	merB	mco -plasmid	copA2 - plasmid	copA2 -SCC	mco -SCC	arsA	arsD	arsD2	arsB		
										arsB (SCC)	arsB (chromos.)	arsB (chromos.- argenteus)
	mercury resistance operon		Multi copper oxidase	Copper exporting ATPase	Copper exporting ATPase	Multi copper oxidase	arsenical pump-driving ATPase	Putative dehydrogenase	trans-acting repressor of arsenic resistance operon	arsenical pump membrane protein		
V40-124624	NEG	NEG	NEG	NEG	NEG	NEG	NEG	NEG	NEG	NEG	POS	NEG
V40-124624: in silico prediction	NEG	NEG	NEG	NEG	NEG	NEG	NEG	NEG	NEG	NEG	POS	NEG
SA-120: in silico prediction	NEG	NEG	NEG	NEG	NEG	NEG	NEG	NEG	NEG	NEG	POS	NEG
61908: in silico prediction	NEG	NEG	NEG	NEG	NEG	NEG	NEG	NEG	NEG	NEG	POS	NEG
M1790_98_1: in silico prediction	NEG	NEG	NEG	NEG	NEG	NEG	NEG	NEG	NEG	NEG	POS	NEG
GKP138-13: in silico prediction	NEG	NEG	NEG	NEG	NEG	NEG	NEG	NEG	NEG	NEG	POS	NEG
GKP138-2: in silico prediction	NEG	NEG	NEG	NEG	NEG	NEG	NEG	NEG	NEG	NEG	POS	NEG
GKP138-31: in silico prediction	NEG	NEG	NEG	NEG	NEG	NEG	NEG	NEG	NEG	NEG	POS	NEG
GKP138-33: in silico prediction	NEG	NEG	NEG	NEG	NEG	NEG	NEG	NEG	NEG	NEG	POS	NEG
GKP138-52: in silico prediction	NEG	NEG	NEG	NEG	NEG	NEG	NEG	NEG	NEG	NEG	POS	NEG
GKP138-78: in silico prediction	NEG	NEG	NEG	NEG	NEG	NEG	NEG	NEG	NEG	NEG	POS	NEG
GKP136-4: in silico prediction	NEG	NEG	NEG	NEG	NEG	NEG	NEG	NEG	NEG	NEG	POS	NEG
ZTA09_03668_9_HSA: in silico prediction	NEG	NEG	NEG	NEG	NEG	NEG	NEG	NEG	NEG	NEG	POS	NEG
GKP136-58: in silico prediction	NEG	NEG	NEG	NEG	NEG	NEG	NEG	NEG	NEG	NEG	POS	NEG
004_0004_23k: in silico prediction	NEG	NEG	NEG	NEG	NEG	NEG	NEG	NEG	NEG	NEG	POS	NEG
BA06_02038: in silico prediction	NEG	NEG	NEG	NEG	NEG	NEG	NEG	NEG	NEG	NEG	POS	NEG
GKP138-4: in silico prediction	NEG	NEG	NEG	NEG	NEG	NEG	NEG	NEG	NEG	NEG	POS	NEG
ZTA10_02421_9_HSA: in silico prediction	NEG	NEG	NEG	NEG	NEG	NEG	NEG	NEG	NEG	NEG	POS	NEG
GKP136-62: in silico prediction	NEG	NEG	NEG	NEG	NEG	NEG	NEG	NEG	NEG	NEG	POS	NEG
LGA251: in silico prediction	NEG	NEG	NEG	NEG	NEG	AMB	NEG	NEG	NEG	POS	POS	NEG
UK_NCTC13552: in silico prediction	NEG	NEG	NEG	NEG	NEG	AMB	NEG	NEG	NEG	POS	POS	NEG
GKP138-71: in silico prediction	NEG	NEG	NEG	NEG	NEG	AMB	NEG	NEG	NEG	POS	POS	NEG
cat-mandible-2013: in silico prediction	NEG	NEG	NEG	NEG	NEG	AMB	NEG	NEG	NEG	POS	NEG	NEG
10_7550_D: in silico prediction	NEG	NEG	NEG	NEG	NEG	AMB	NEG	NEG	NEG	POS	POS	NEG
H114440275_A: in silico prediction	NEG	NEG	NEG	NEG	NEG	AMB	NEG	NEG	NEG	POS	POS	NEG

STRAIN / ISOLATE	HEAVY METAL RESISTANCES, SCCmec AND OTHERS										RESISTANCE : PENICILLINASE		
	arsC		czrB	czrC	cadA	cadC	cadD		cadX (plasmid)	cadX (SCC)	blaZ	blaI	blaR
	arsC (chromos.)	arsC (SCC or plasmidic)					cadD (total)	cadD (R35)					
	arsenate reductase		zink and cobalt transporter protein	cadmium and zinc resistance gene C, heavy metal translocating P-type ATPase. Frequently associated with SCCmec elements from livestock MRSA	cadmium transporting ATPase	putative regulator of cadmium efflux	cadmium transport protein D		Putative regulator of cadmium efflux		beta-lactamase	beta lactamase repressor (inhibitor)	beta-lactamase regulatory protein
V40-124624	POS	NEG	POS	NEG	NEG	NEG	NEG	NEG	NEG	NEG	NEG	NEG	NEG
V40-124624: in silico prediction	POS	NEG	POS	NEG	NEG	NEG	NEG	NEG	NEG	NEG	NEG	NEG	NEG
SA-120: in silico prediction	POS	NEG	POS	NEG	NEG	NEG	NEG	NEG	NEG	NEG	NEG	NEG	NEG
61908: in silico prediction	POS	NEG	POS	NEG	NEG	NEG	NEG	NEG	NEG	NEG	NEG	NEG	NEG
M1790_98_1: in silico prediction	POS	NEG	POS	NEG	NEG	NEG	NEG	NEG	NEG	NEG	NEG	NEG	NEG
GKP138-13: in silico prediction	POS	NEG	POS	NEG	NEG	NEG	NEG	NEG	NEG	NEG	NEG	NEG	NEG
GKP138-2: in silico prediction	POS	NEG	POS	NEG	NEG	NEG	NEG	NEG	NEG	NEG	NEG	NEG	NEG
GKP138-31: in silico prediction	POS	NEG	POS	NEG	NEG	NEG	NEG	NEG	NEG	NEG	NEG	NEG	NEG
GKP138-33: in silico prediction	POS	NEG	POS	NEG	NEG	NEG	NEG	NEG	NEG	NEG	NEG	NEG	NEG
GKP138-52: in silico prediction	POS	NEG	POS	NEG	NEG	NEG	NEG	NEG	NEG	NEG	NEG	NEG	NEG
GKP138-78: in silico prediction	POS	NEG	POS	NEG	NEG	NEG	NEG	NEG	NEG	NEG	NEG	NEG	NEG
GKP136-4: in silico prediction	POS	NEG	POS	NEG	NEG	NEG	NEG	NEG	NEG	NEG	NEG	NEG	NEG
ZTA09_03668_9_HSA: in silico prediction	POS	NEG	POS	NEG	NEG	NEG	NEG	NEG	NEG	NEG	NEG	NEG	NEG
GKP136-58: in silico prediction	POS	NEG	POS	NEG	NEG	NEG	NEG	NEG	NEG	NEG	POS	POS	AMB
004_0004_23k: in silico prediction	POS	NEG	POS	NEG	NEG	NEG	NEG	NEG	NEG	NEG	POS	POS	AMB
BA06_02038: in silico prediction	POS	NEG	POS	NEG	NEG	NEG	NEG	NEG	NEG	NEG	POS	POS	AMB
GKP138-4: in silico prediction	POS	NEG	POS	NEG	POS	POS	NEG	POS	NEG	NEG	NEG	NEG	NEG
ZTA10_02421_9_HSA: in silico prediction	POS	NEG	POS	NEG	POS	POS	NEG	POS	NEG	NEG	NEG	NEG	NEG
GKP136-62: in silico prediction	POS	NEG	POS	NEG	NEG	NEG	POS	NEG	POS	NEG	NEG	NEG	NEG
LGA251: in silico prediction	POS	POS	POS	NEG	NEG	NEG	NEG	NEG	NEG	NEG	NEG	NEG	NEG
UK_NCTC13552: in silico prediction	POS	POS	POS	NEG	NEG	NEG	NEG	NEG	NEG	NEG	NEG	NEG	NEG
GKP138-71: in silico prediction	POS	POS	POS	NEG	NEG	NEG	NEG	NEG	NEG	NEG	NEG	NEG	NEG
cat-mandible-2013: in silico prediction	POS	POS	POS	NEG	NEG	NEG	NEG	NEG	NEG	NEG	NEG	NEG	NEG
10_7550_D: in silico prediction	POS	POS	POS	NEG	NEG	NEG	NEG	NEG	NEG	NEG	NEG	NEG	NEG
H114440275_A: in silico prediction	POS	POS	POS	NEG	NEG	NEG	NEG	NEG	NEG	NEG	NEG	NEG	NEG

STRAIN / ISOLATE	RESISTANCE : MLS-ANTIBIOTICS																
	ermA	ermA-43	ermB	ermC		ermF	ermT	linA/lnuA	lnuB	lsa-B	lsa-E	msrA	mefA	mph(C)	vat(A)	vat(B)	vga(A)
				ermC	ermC-GM												
	rRNA adenine N-6-methyl-transferase, erythromycin/clindamycin resistance		erythro-mycin/clindamycin resistance	rRNA adenine N-6-methyltrans-ferase	rRNA adenine methylase-like protein ErmGM	rRNA adenine N-6-methyltrans- ferase	rRNA adenine N-6-methyltrans- ferase	Lincosamid-Nucleotidyltransferase	lincosamide nucleotidyltransferase	lincosamide ABC transporter	lincosamide ABC transporter	energy-dependent efflux of erythro-mycin	macrolide efflux protein A	probable lysylphos-phatidyl-glycerol synthetase	virginiamycin A acetyltrans- ferase	acetyl-transferase inactivating streptogramin A	ATP binding protein, streptogramin-A-resistance
V40-124624	NEG	NEG	NEG	NEG	NEG	NEG	NEG	NEG	NEG	NEG	NEG	NEG	NEG	NEG	NEG	NEG	NEG
V40-124624: in silico prediction	NEG	NEG	NEG	NEG	NEG	NEG	NEG	NEG	NEG	NEG	NEG	NEG	NEG	NEG	NEG	NEG	NEG
SA-120: in silico prediction	NEG	NEG	NEG	NEG	NEG	NEG	NEG	NEG	NEG	NEG	NEG	NEG	NEG	NEG	NEG	NEG	NEG
61908: in silico prediction	NEG	NEG	NEG	NEG	NEG	NEG	NEG	NEG	NEG	NEG	NEG	NEG	NEG	NEG	NEG	NEG	NEG
M1790_98_1: in silico prediction	NEG	NEG	NEG	NEG	NEG	NEG	NEG	NEG	NEG	NEG	NEG	NEG	NEG	NEG	NEG	NEG	NEG
GKP138-13: in silico prediction	NEG	NEG	NEG	NEG	NEG	NEG	NEG	NEG	NEG	NEG	NEG	NEG	NEG	NEG	NEG	NEG	NEG
GKP138-2: in silico prediction	NEG	NEG	NEG	NEG	NEG	NEG	NEG	NEG	NEG	NEG	NEG	NEG	NEG	NEG	NEG	NEG	NEG
GKP138-31: in silico prediction	NEG	NEG	NEG	NEG	NEG	NEG	NEG	NEG	NEG	NEG	NEG	NEG	NEG	NEG	NEG	NEG	NEG
GKP138-33: in silico prediction	NEG	NEG	NEG	NEG	NEG	NEG	NEG	NEG	NEG	NEG	NEG	NEG	NEG	NEG	NEG	NEG	NEG
GKP138-52: in silico prediction	NEG	NEG	NEG	NEG	NEG	NEG	NEG	NEG	NEG	NEG	NEG	NEG	NEG	NEG	NEG	NEG	NEG
GKP138-78: in silico prediction	NEG	NEG	NEG	NEG	NEG	NEG	NEG	NEG	NEG	NEG	NEG	NEG	NEG	NEG	NEG	NEG	NEG
GKP136-4: in silico prediction	NEG	NEG	NEG	NEG	NEG	NEG	NEG	NEG	NEG	NEG	NEG	NEG	NEG	NEG	NEG	NEG	NEG
ZTA09_03668_9_HSA: in silico prediction	NEG	NEG	NEG	NEG	NEG	NEG	NEG	NEG	NEG	NEG	NEG	NEG	NEG	NEG	NEG	NEG	NEG
GKP136-58: in silico prediction	NEG	NEG	NEG	NEG	NEG	NEG	NEG	NEG	NEG	NEG	NEG	NEG	NEG	NEG	NEG	NEG	NEG
004_0004_23k: in silico prediction	NEG	NEG	NEG	NEG	NEG	NEG	NEG	NEG	NEG	NEG	NEG	NEG	NEG	NEG	NEG	NEG	NEG
BA06_02038: in silico prediction	NEG	NEG	NEG	NEG	NEG	NEG	NEG	NEG	NEG	NEG	NEG	NEG	NEG	NEG	NEG	NEG	NEG
GKP138-4: in silico prediction	NEG	NEG	NEG	NEG	NEG	NEG	NEG	NEG	NEG	NEG	NEG	NEG	NEG	NEG	NEG	NEG	NEG
ZTA10_02421_9_HSA: in silico prediction	NEG	NEG	NEG	NEG	NEG	NEG	NEG	NEG	NEG	NEG	NEG	NEG	NEG	NEG	NEG	NEG	NEG
GKP136-62: in silico prediction	NEG	NEG	NEG	NEG	NEG	NEG	NEG	NEG	NEG	NEG	NEG	NEG	NEG	NEG	NEG	NEG	NEG
LGA251: in silico prediction	NEG	NEG	NEG	NEG	NEG	NEG	NEG	NEG	NEG	NEG	NEG	NEG	NEG	NEG	NEG	NEG	NEG
UK_NCTC13552: in silico prediction	NEG	NEG	NEG	NEG	NEG	NEG	NEG	NEG	NEG	NEG	NEG	NEG	NEG	NEG	NEG	NEG	NEG
GKP138-71: in silico prediction	NEG	NEG	NEG	NEG	NEG	NEG	NEG	NEG	NEG	NEG	NEG	NEG	NEG	NEG	NEG	NEG	NEG
cat-mandible-2013: in silico prediction	NEG	NEG	NEG	NEG	NEG	NEG	NEG	NEG	NEG	NEG	NEG	NEG	NEG	NEG	NEG	NEG	NEG
10_7550_D: in silico prediction	NEG	NEG	NEG	NEG	NEG	NEG	NEG	NEG	NEG	NEG	NEG	NEG	NEG	NEG	NEG	NEG	NEG
H114440275_A: in silico prediction	NEG	NEG	NEG	NEG	NEG	NEG	NEG	NEG	NEG	NEG	NEG	NEG	NEG	NEG	NEG	NEG	NEG

STRAIN / ISOLATE	RESISTANCE : MLS-ANTIBIOTICS						RESISTANCE : AMINOGLYSOSIDES								
	vga(A) (BM 3327)	vgaB	vgaC	vgaD	vgaE	vgb	aacA-aphD	aadD	ant9		ble	aad6	aadA	aadE	aphA3
									ant9-C2944	ant9					
		streptogramin A ABC	streptogramin A ABC transporter		streptogramin A ABC	virginia-mycin B hydrolase	bifunctional enzyme Aac/Aph, gentamicin resistance	amino-glycoside adenyl-transferase,tobramycin resistance	adenyltransferase AAd9		bleomycin resistance protein	aminoglycoside 6-adenylyltransferase	aminoglycoside-3'-adenylyltransferase	streptomycin aminoglycoside 6-adenyltransferase	3'5'-aminog-lycoside phospho-transferase, neo-/ kanamycin resistance
V40-124624	NEG	NEG	NEG	NEG	NEG	NEG	NEG	NEG	NEG	NEG	NEG	NEG	NEG	NEG	NEG
V40-124624: in silico prediction	NEG	NEG	NEG	NEG	NEG	NEG	NEG	NEG	NEG	NEG	NEG	NEG	NEG	NEG	NEG
SA-120: in silico prediction	NEG	NEG	NEG	NEG	NEG	NEG	NEG	NEG	NEG	NEG	NEG	NEG	NEG	NEG	NEG
61908: in silico prediction	NEG	NEG	NEG	NEG	NEG	NEG	NEG	NEG	NEG	NEG	NEG	NEG	NEG	NEG	NEG
M1790_98_1: in silico prediction	NEG	NEG	NEG	NEG	NEG	NEG	NEG	NEG	NEG	NEG	NEG	NEG	NEG	NEG	NEG
GKP138-13: in silico prediction	NEG	NEG	NEG	NEG	NEG	NEG	NEG	NEG	NEG	NEG	NEG	NEG	NEG	NEG	NEG
GKP138-2: in silico prediction	NEG	NEG	NEG	NEG	NEG	NEG	NEG	NEG	NEG	NEG	NEG	NEG	NEG	NEG	NEG
GKP138-31: in silico prediction	NEG	NEG	NEG	NEG	NEG	NEG	NEG	NEG	NEG	NEG	NEG	NEG	NEG	NEG	NEG
GKP138-33: in silico prediction	NEG	NEG	NEG	NEG	NEG	NEG	NEG	NEG	NEG	NEG	NEG	NEG	NEG	NEG	NEG
GKP138-52: in silico prediction	NEG	NEG	NEG	NEG	NEG	NEG	NEG	NEG	NEG	NEG	NEG	NEG	NEG	NEG	NEG
GKP138-78: in silico prediction	NEG	NEG	NEG	NEG	NEG	NEG	NEG	NEG	NEG	NEG	NEG	NEG	NEG	NEG	NEG
GKP136-4: in silico prediction	NEG	NEG	NEG	NEG	NEG	NEG	NEG	NEG	NEG	NEG	NEG	NEG	NEG	NEG	NEG
ZTA09_03668_9_HSA: in silico prediction	NEG	NEG	NEG	NEG	NEG	NEG	NEG	NEG	NEG	NEG	NEG	NEG	NEG	NEG	NEG
GKP136-58: in silico prediction	NEG	NEG	NEG	NEG	NEG	NEG	NEG	NEG	NEG	NEG	NEG	NEG	NEG	NEG	NEG
004_0004_23k: in silico prediction	NEG	NEG	NEG	NEG	NEG	NEG	NEG	NEG	NEG	NEG	NEG	NEG	NEG	NEG	NEG
BA06_02038: in silico prediction	NEG	NEG	NEG	NEG	NEG	NEG	NEG	NEG	NEG	NEG	NEG	NEG	NEG	NEG	NEG
GKP138-4: in silico prediction	NEG	NEG	NEG	NEG	NEG	NEG	NEG	NEG	NEG	NEG	NEG	NEG	NEG	NEG	NEG
ZTA10_02421_9_HSA: in silico prediction	NEG	NEG	NEG	NEG	NEG	NEG	NEG	NEG	NEG	NEG	NEG	NEG	NEG	NEG	NEG
GKP136-62: in silico prediction	NEG	NEG	NEG	NEG	NEG	NEG	NEG	NEG	NEG	NEG	NEG	NEG	NEG	NEG	NEG
LGA251: in silico prediction	NEG	NEG	NEG	NEG	NEG	NEG	NEG	NEG	NEG	NEG	NEG	NEG	NEG	NEG	NEG
UK_NCTC13552: in silico prediction	NEG	NEG	NEG	NEG	NEG	NEG	NEG	NEG	NEG	NEG	NEG	NEG	NEG	NEG	NEG
GKP138-71: in silico prediction	NEG	NEG	NEG	NEG	NEG	NEG	NEG	NEG	NEG	NEG	NEG	NEG	NEG	NEG	NEG
cat-mandible-2013: in silico prediction	NEG	NEG	NEG	NEG	NEG	NEG	NEG	NEG	NEG	NEG	NEG	NEG	NEG	NEG	NEG
10_7550_D: in silico prediction	NEG	NEG	NEG	NEG	NEG	NEG	NEG	NEG	NEG	NEG	NEG	NEG	NEG	NEG	NEG
H114440275_A: in silico prediction	NEG	NEG	NEG	NEG	NEG	NEG	NEG	NEG	NEG	NEG	NEG	NEG	NEG	NEG	NEG

STRAIN / ISOLATE	RESISTANCE : MISCELLANEOUS GENES				RESISTANCE : MISCELLANEOUS GENES																
									tetM												
	sat	dfrA	dfrG	far1	mupA	mupB	tetK	tetL	tetM	tetM-O	tetM-S	cat	cfr	fexA	apmA	fosB	qacA	qacC	smr	vanA ,vanB, vanZ
	strepto-thricine-acetyl-transferase	dihydro-folate reductase type 1	dihydrofolate reductase	fusidic acid resistence	mupirocin resistence protein	plasmidic isoleucyl-tR	tetracycline resistance	Tetracycline resistanc	tetracycline resistance			chloramphenicol acetyltransferase	23S rRNA methyltransferase	chloramphenicol/florfenicol exporter	aminocyclitol acetyltr	metallothiol transferase	quaternary ammonium compound resistance protein A	quaternary ammonium compound resistance protein C		vancomycin resistance genes
V40-124624	NEG	NEG	NEG	NEG	NEG	NEG	NEG	NEG	NEG	NEG	NEG	NEG	NEG	NEG	NEG	POS	NEG	NEG	NEG	NEG
V40-124624: in silico prediction	NEG	NEG	NEG	NEG	NEG	NEG	NEG	NEG	NEG	NEG	NEG	NEG	NEG	NEG	NEG	POS	NEG	NEG	NEG	NEG
SA-120: in silico prediction	NEG	NEG	NEG	NEG	NEG	NEG	NEG	NEG	NEG	NEG	NEG	NEG	NEG	NEG	NEG	POS	NEG	NEG	NEG	NEG
61908: in silico prediction	NEG	NEG	NEG	NEG	NEG	NEG	NEG	NEG	NEG	NEG	NEG	NEG	NEG	NEG	NEG	POS	NEG	NEG	NEG	NEG
M1790_98_1: in silico prediction	NEG	NEG	NEG	NEG	NEG	NEG	NEG	NEG	NEG	NEG	NEG	NEG	NEG	NEG	NEG	POS	NEG	NEG	NEG	NEG
GKP138-13: in silico prediction	NEG	NEG	NEG	NEG	NEG	NEG	NEG	NEG	NEG	NEG	NEG	NEG	NEG	NEG	NEG	POS	NEG	NEG	NEG	NEG
GKP138-2: in silico prediction	NEG	NEG	NEG	NEG	NEG	NEG	NEG	NEG	NEG	NEG	NEG	NEG	NEG	NEG	NEG	POS	NEG	NEG	NEG	NEG
GKP138-31: in silico prediction	NEG	NEG	NEG	NEG	NEG	NEG	NEG	NEG	NEG	NEG	NEG	NEG	NEG	NEG	NEG	POS	NEG	NEG	NEG	NEG
GKP138-33: in silico prediction	NEG	NEG	NEG	NEG	NEG	NEG	NEG	NEG	NEG	NEG	NEG	NEG	NEG	NEG	NEG	POS	NEG	NEG	NEG	NEG
GKP138-52: in silico prediction	NEG	NEG	NEG	NEG	NEG	NEG	NEG	NEG	NEG	NEG	NEG	NEG	NEG	NEG	NEG	POS	NEG	NEG	NEG	NEG
GKP138-78: in silico prediction	NEG	NEG	NEG	NEG	NEG	NEG	NEG	NEG	NEG	NEG	NEG	NEG	NEG	NEG	NEG	POS	NEG	NEG	NEG	NEG
GKP136-4: in silico prediction	NEG	NEG	NEG	NEG	NEG	NEG	NEG	NEG	NEG	NEG	NEG	NEG	NEG	NEG	NEG	POS	NEG	NEG	NEG	NEG
ZTA09_03668_9_HSA: in silico prediction	NEG	NEG	NEG	NEG	NEG	NEG	NEG	NEG	NEG	NEG	NEG	NEG	NEG	NEG	NEG	POS	NEG	NEG	NEG	NEG
GKP136-58: in silico prediction	NEG	NEG	NEG	NEG	NEG	NEG	NEG	NEG	NEG	NEG	NEG	NEG	NEG	NEG	NEG	POS	NEG	NEG	NEG	NEG
004_0004_23k: in silico prediction	NEG	NEG	NEG	NEG	NEG	NEG	POS	NEG	NEG	NEG	NEG	NEG	NEG	NEG	NEG	POS	NEG	NEG	NEG	NEG
BA06_02038: in silico prediction	NEG	NEG	NEG	NEG	NEG	NEG	POS	NEG	NEG	NEG	NEG	NEG	NEG	NEG	NEG	POS	NEG	NEG	NEG	NEG
GKP138-4: in silico prediction	NEG	NEG	NEG	NEG	NEG	NEG	NEG	NEG	NEG	NEG	NEG	NEG	NEG	NEG	NEG	POS	NEG	NEG	NEG	NEG
ZTA10_02421_9_HSA: in silico prediction	NEG	NEG	NEG	NEG	NEG	NEG	NEG	NEG	NEG	NEG	NEG	NEG	NEG	NEG	NEG	POS	NEG	NEG	NEG	NEG
GKP136-62: in silico prediction	NEG	NEG	NEG	NEG	NEG	NEG	NEG	NEG	NEG	NEG	NEG	NEG	NEG	NEG	NEG	POS	NEG	NEG	NEG	NEG
LGA251: in silico prediction	NEG	NEG	NEG	NEG	NEG	NEG	NEG	NEG	NEG	NEG	NEG	NEG	NEG	NEG	NEG	POS	NEG	NEG	NEG	NEG
UK_NCTC13552: in silico prediction	NEG	NEG	NEG	NEG	NEG	NEG	NEG	NEG	NEG	NEG	NEG	NEG	NEG	NEG	NEG	POS	NEG	NEG	NEG	NEG
GKP138-71: in silico prediction	NEG	NEG	NEG	NEG	NEG	NEG	NEG	NEG	NEG	NEG	NEG	NEG	NEG	NEG	NEG	POS	NEG	NEG	NEG	NEG
cat-mandible-2013: in silico prediction	NEG	NEG	NEG	NEG	NEG	NEG	NEG	NEG	NEG	NEG	NEG	NEG	NEG	NEG	NEG	POS	NEG	NEG	NEG	NEG
10_7550_D: in silico prediction	NEG	NEG	NEG	NEG	NEG	NEG	NEG	NEG	NEG	NEG	NEG	NEG	NEG	NEG	NEG	POS	NEG	NEG	NEG	NEG
H114440275_A: in silico prediction	NEG	NEG	NEG	NEG	NEG	NEG	NEG	NEG	NEG	NEG	NEG	NEG	NEG	NEG	NEG	POS	NEG	NEG	NEG	NEG

STRAIN / ISOLATE	VIRULENCE : ENTEROTOXINS and TOX.SHOCK TOXIN																			
	tst1	seA			seB	seC	seD	seD2	seE	seH	seJ	seK	seL	seN2	seQ	seR	seS	seT	seU2	seW
	tst1 (consensus)	entA	entA (320E)	entA (N315) / entP																
	toxic shock syndrome toxin 1	Enterotoxin A	Enterotoxin A, allele from strain 320E	Enterotoxin A, allele from strain N315 = Entero-toxin P	Enterotoxin B	Enterotoxin C	Enterotoxin D	Enterotoxin similar to EntD	Enterotoxin E	Enterotoxin H	Enterotoxin J	Enterotoxin K	Enterotoxin L	Putative Enterotoxin N2	Enterotoxin Q	Enterotoxin R	Enterotoxin S	Enterotoxin T	Enterotoxin U2	Putative Enterotoxin
V40-124624	NEG	NEG	NEG	NEG	NEG	NEG	NEG	NEG	POS	NEG	NEG	NEG	NEG	NEG	NEG	NEG	NEG	NEG	NEG	NEG
V40-124624: in silico prediction	NEG	NEG	NEG	NEG	NEG	NEG	NEG	NEG	POS	NEG	NEG	NEG	NEG	NEG	NEG	NEG	NEG	NEG	NEG	NEG
SA-120: in silico prediction	NEG	NEG	NEG	NEG	NEG	NEG	NEG	NEG	POS	NEG	NEG	NEG	NEG	NEG	NEG	NEG	NEG	NEG	NEG	NEG
61908: in silico prediction	NEG	NEG	NEG	NEG	NEG	NEG	NEG	NEG	NEG	NEG	NEG	NEG	NEG	NEG	NEG	NEG	NEG	NEG	NEG	NEG
M1790_98_1: in silico prediction	NEG	NEG	NEG	NEG	NEG	NEG	NEG	NEG	NEG	NEG	NEG	NEG	NEG	NEG	NEG	NEG	NEG	NEG	NEG	NEG
GKP138-13: in silico prediction	NEG	NEG	NEG	NEG	NEG	NEG	NEG	NEG	NEG	NEG	NEG	NEG	NEG	NEG	NEG	NEG	NEG	NEG	NEG	NEG
GKP138-2: in silico prediction	NEG	NEG	NEG	NEG	NEG	NEG	NEG	NEG	NEG	NEG	NEG	NEG	NEG	NEG	NEG	NEG	NEG	NEG	NEG	NEG
GKP138-31: in silico prediction	NEG	NEG	NEG	NEG	NEG	NEG	NEG	NEG	NEG	NEG	NEG	NEG	NEG	NEG	NEG	NEG	NEG	NEG	NEG	NEG
GKP138-33: in silico prediction	NEG	NEG	NEG	NEG	NEG	NEG	NEG	NEG	NEG	NEG	NEG	NEG	NEG	NEG	NEG	NEG	NEG	NEG	NEG	NEG
GKP138-52: in silico prediction	NEG	NEG	NEG	NEG	NEG	NEG	NEG	NEG	NEG	NEG	NEG	NEG	NEG	NEG	NEG	NEG	NEG	NEG	NEG	NEG
GKP138-78: in silico prediction	NEG	NEG	NEG	NEG	NEG	NEG	NEG	NEG	NEG	NEG	NEG	NEG	NEG	NEG	NEG	NEG	NEG	NEG	NEG	NEG
GKP136-4: in silico prediction	NEG	NEG	NEG	NEG	NEG	NEG	NEG	NEG	NEG	NEG	NEG	NEG	NEG	NEG	NEG	NEG	NEG	NEG	NEG	NEG
ZTA09_03668_9_HSA: in silico prediction	NEG	NEG	NEG	NEG	NEG	NEG	NEG	NEG	NEG	NEG	NEG	NEG	NEG	NEG	NEG	NEG	NEG	NEG	NEG	NEG
GKP136-58: in silico prediction	POS	NEG	NEG	NEG	AMB	POS	NEG	NEG	NEG	NEG	NEG	NEG	POS	NEG	NEG	NEG	NEG	NEG	NEG	NEG
004_0004_23k: in silico prediction	POS	NEG	NEG	NEG	AMB	POS	NEG	NEG	NEG	NEG	NEG	NEG	POS	NEG	NEG	NEG	NEG	NEG	NEG	NEG
BA06_02038: in silico prediction	POS	NEG	NEG	NEG	AMB	POS	NEG	NEG	NEG	NEG	NEG	NEG	POS	NEG	NEG	NEG	NEG	NEG	NEG	NEG
GKP138-4: in silico prediction	NEG	NEG	NEG	NEG	NEG	NEG	NEG	NEG	NEG	NEG	NEG	NEG	NEG	NEG	NEG	NEG	NEG	NEG	NEG	NEG
ZTA10_02421_9_HSA: in silico prediction	NEG	NEG	NEG	NEG	NEG	NEG	NEG	NEG	NEG	NEG	NEG	NEG	NEG	NEG	NEG	NEG	NEG	NEG	NEG	NEG
GKP136-62: in silico prediction	NEG	NEG	NEG	NEG	NEG	NEG	NEG	NEG	NEG	NEG	NEG	NEG	NEG	NEG	NEG	NEG	NEG	NEG	NEG	NEG
LGA251: in silico prediction	NEG	NEG	NEG	NEG	NEG	NEG	NEG	NEG	NEG	NEG	NEG	NEG	NEG	NEG	NEG	NEG	NEG	NEG	NEG	NEG
UK_NCTC13552: in silico prediction	NEG	NEG	NEG	NEG	NEG	NEG	NEG	NEG	NEG	NEG	NEG	NEG	NEG	NEG	NEG	NEG	NEG	NEG	NEG	NEG
GKP138-71: in silico prediction	NEG	NEG	NEG	NEG	NEG	NEG	NEG	NEG	NEG	NEG	NEG	NEG	NEG	NEG	NEG	NEG	NEG	NEG	NEG	NEG
cat-mandible-2013: in silico prediction	NEG	NEG	NEG	NEG	NEG	NEG	NEG	NEG	NEG	NEG	NEG	NEG	NEG	NEG	NEG	NEG	NEG	NEG	NEG	NEG
10_7550_D: in silico prediction	NEG	NEG	NEG	NEG	NEG	NEG	NEG	NEG	NEG	NEG	NEG	NEG	NEG	NEG	NEG	NEG	NEG	NEG	NEG	NEG
H114440275_A: in silico prediction	NEG	NEG	NEG	NEG	NEG	NEG	NEG	NEG	NEG	NEG	NEG	NEG	NEG	NEG	NEG	NEG	NEG	NEG	NEG	NEG

STRAIN / ISOLATE	VIRULENCE : ENTEROTOXINS								VIRULENCE : HLG AND LEUKOCIDINS													
	egc (total)	selg	seli	selm	sen	selo	selu	ORF CM14	lukF	lukS		hlgA	lukF/S (int)	lukF-PV	lukS-PV	lukF-PV (P83)	lukM	lukD	lukE	lukX	lukY	
										lukS	lukS (ST22+ST45)										lukY	lukY (ST30+ST45)
	egc cluster	Enterotoxin G	Enterotoxin I	Enterotoxin M	Enterotoxin N	Enterotoxin O	Enterotoxin U and/or Y	Enterotoxin-like protein ORF CM14	Haemolysin gamma / leukocidin, component B	Haemolysin gamma / leukocidin, component C		Haemolysin gamma,	intermedius group leukocidin	Panton Valentine leukocidin F component	Panton Valentine leukocidin S component	F component from ruminant leukocidin	S component from ruminant leukocidin	leukocidin D component	leukocidin E component	leukocidin/ haemolysin toxin family protein	leukocidin/haemolysin toxin family protein	
V40-124624	NEG	NEG	NEG	NEG	NEG	NEG	NEG	NEG	POS	POS	AMB	POS	NEG	NEG	NEG	NEG	NEG	POS	NEG	POS	POS	NEG
V40-124624: in silico prediction	NEG	NEG	NEG	NEG	NEG	NEG	NEG	NEG	POS	POS	AMB	POS	NEG	NEG	NEG	NEG	NEG	POS	AMB	POS	POS	NEG
SA-120: in silico prediction	NEG	NEG	NEG	NEG	NEG	NEG	NEG	NEG	POS	POS	AMB	POS	NEG	NEG	NEG	NEG	NEG	POS	AMB	POS	POS	NEG
61908: in silico prediction	NEG	NEG	NEG	NEG	NEG	NEG	NEG	NEG	POS	POS	AMB	POS	NEG	NEG	NEG	NEG	NEG	POS	AMB	POS	POS	NEG
M1790_98_1: in silico prediction	NEG	NEG	NEG	NEG	NEG	NEG	NEG	NEG	POS	POS	AMB	POS	NEG	NEG	NEG	NEG	NEG	POS	AMB	POS	POS	NEG
GKP138-13: in silico prediction	NEG	NEG	NEG	NEG	NEG	NEG	NEG	NEG	POS	POS	AMB	POS	NEG	NEG	NEG	NEG	NEG	POS	AMB	POS	POS	NEG
GKP138-2: in silico prediction	NEG	NEG	NEG	NEG	NEG	NEG	NEG	NEG	POS	POS	AMB	POS	NEG	NEG	NEG	NEG	NEG	POS	AMB	POS	POS	NEG
GKP138-31: in silico prediction	NEG	NEG	NEG	NEG	NEG	NEG	NEG	NEG	POS	POS	AMB	POS	NEG	NEG	NEG	NEG	NEG	POS	AMB	POS	POS	NEG
GKP138-33: in silico prediction	NEG	NEG	NEG	NEG	NEG	NEG	NEG	NEG	POS	POS	AMB	POS	NEG	NEG	NEG	NEG	NEG	POS	AMB	POS	POS	NEG
GKP138-52: in silico prediction	NEG	NEG	NEG	NEG	NEG	NEG	NEG	NEG	POS	POS	AMB	POS	NEG	NEG	NEG	NEG	NEG	POS	AMB	POS	POS	NEG
GKP138-78: in silico prediction	NEG	NEG	NEG	NEG	NEG	NEG	NEG	NEG	POS	POS	AMB	POS	NEG	NEG	NEG	NEG	NEG	POS	AMB	POS	POS	NEG
GKP136-4: in silico prediction	NEG	NEG	NEG	NEG	NEG	NEG	NEG	NEG	POS	POS	AMB	POS	NEG	NEG	NEG	NEG	NEG	POS	AMB	POS	POS	NEG
ZTA09_03668_9_HSA: in silico prediction	NEG	NEG	NEG	NEG	NEG	NEG	NEG	NEG	POS	POS	AMB	POS	NEG	NEG	NEG	NEG	NEG	POS	AMB	POS	POS	NEG
GKP136-58: in silico prediction	NEG	NEG	NEG	NEG	NEG	NEG	NEG	NEG	POS	POS	AMB	POS	NEG	NEG	NEG	NEG	NEG	POS	AMB	POS	POS	NEG
004_0004_23k: in silico prediction	NEG	NEG	NEG	NEG	NEG	NEG	NEG	NEG	POS	POS	AMB	POS	NEG	NEG	NEG	NEG	NEG	POS	AMB	POS	POS	NEG
BA06_02038: in silico prediction	NEG	NEG	NEG	NEG	NEG	NEG	NEG	NEG	POS	POS	AMB	POS	NEG	NEG	NEG	NEG	NEG	POS	AMB	POS	POS	NEG
GKP138-4: in silico prediction	NEG	NEG	NEG	NEG	NEG	NEG	NEG	NEG	POS	POS	AMB	POS	NEG	NEG	NEG	NEG	NEG	POS	AMB	POS	POS	NEG
ZTA10_02421_9_HSA: in silico prediction	NEG	NEG	NEG	NEG	NEG	NEG	NEG	NEG	POS	POS	AMB	POS	NEG	NEG	NEG	NEG	NEG	POS	AMB	POS	POS	NEG
GKP136-62: in silico prediction	NEG	NEG	NEG	NEG	NEG	NEG	NEG	NEG	POS	POS	AMB	POS	NEG	NEG	NEG	NEG	NEG	POS	AMB	POS	POS	NEG
LGA251: in silico prediction	NEG	NEG	NEG	NEG	NEG	NEG	NEG	NEG	POS	POS	AMB	POS	NEG	NEG	NEG	NEG	NEG	POS	AMB	POS	POS	NEG
UK_NCTC13552: in silico prediction	NEG	NEG	NEG	NEG	NEG	NEG	NEG	NEG	POS	POS	AMB	POS	NEG	NEG	NEG	NEG	NEG	POS	AMB	POS	POS	NEG
GKP138-71: in silico prediction	NEG	NEG	NEG	NEG	NEG	NEG	NEG	NEG	POS	POS	AMB	POS	NEG	NEG	NEG	NEG	NEG	POS	AMB	POS	POS	NEG
cat-mandible-2013: in silico prediction	NEG	NEG	NEG	NEG	NEG	NEG	NEG	NEG	POS	POS	AMB	POS	NEG	NEG	NEG	NEG	NEG	POS	AMB	POS	POS	NEG
10_7550_D: in silico prediction	NEG	NEG	NEG	NEG	NEG	NEG	NEG	NEG	POS	POS	AMB	POS	NEG	NEG	NEG	NEG	NEG	POS	AMB	POS	POS	NEG
H114440275_A: in silico prediction	NEG	NEG	NEG	NEG	NEG	NEG	NEG	NEG	POS	POS	AMB	POS	NEG	NEG	NEG	NEG	NEG	POS	AMB	POS	POS	NEG

STRAIN / ISOLATE	VIRULENCE : HAEMOLYSINS								VIRULENCE : HLB-CONV PHAGES			VIRULENCE : OTHER FACTORS								
	corB (=hl)	hla	hlIII		hlb				sak	chp	scn	etA	etB	etD	etE / "etD2"	edinA	edinB	edinC	esxA	esxB
			hlIII (cons)	hlIII (other than RF122)	hlb-probe 1	hlb-probe 2	hlb-probe 3	un-truncated hlb												
	Putative membrane protein	Haemolysin alpha	Putative membrane protein		haemolysin beta				staphylo-kinase	chemotaxis-inhibiting protein (CHIPS)	Staphyl. Comple-ment inhibitor	exfoliative toxin serotype A	exfoliative toxin serotype B	exfoliative toxin D	exfoliative toxin D2	epidermal cell differen-tiation inhibitor	epidermal cell differen-tiation inhibitor B	epidermal cell differen-tiation inhibitor C	virulence factor esxA	virulence factor esxB
V40-124624	POS	POS	POS	NEG	POS	POS	POS	NEG	NEG	NEG	NEG	NEG	NEG	NEG	NEG	NEG	NEG	NEG	POS	NEG
V40-124624: in silico prediction	POS	POS	POS	NEG	POS	POS	POS	POS	NEG	NEG	NEG	NEG	NEG	NEG	NEG	NEG	NEG	NEG	POS	NEG
SA-120: in silico prediction	POS	POS	POS	NEG	POS	POS	POS	POS	NEG	NEG	NEG	NEG	NEG	NEG	NEG	NEG	NEG	NEG	POS	NEG
61908: in silico prediction	POS	POS	POS	NEG	AMB	NEG	AMB	POS	NEG	NEG	NEG	NEG	NEG	NEG	NEG	NEG	NEG	NEG	POS	NEG
M1790_98_1: in silico prediction	POS	POS	POS	NEG	POS	POS	POS	POS	NEG	NEG	NEG	NEG	NEG	NEG	NEG	NEG	NEG	NEG	POS	NEG
GKP138-13: in silico prediction	POS	POS	POS	NEG	POS	POS	POS	POS	NEG	NEG	NEG	NEG	NEG	NEG	NEG	NEG	NEG	NEG	POS	NEG
GKP138-2: in silico prediction	POS	NEG	POS	NEG	POS	POS	POS	POS	NEG	NEG	NEG	NEG	NEG	NEG	NEG	NEG	NEG	NEG	POS	NEG
GKP138-31: in silico prediction	POS	POS	POS	NEG	POS	POS	POS	POS	NEG	NEG	NEG	NEG	NEG	NEG	NEG	NEG	NEG	NEG	POS	NEG
GKP138-33: in silico prediction	POS	POS	POS	NEG	POS	POS	POS	POS	NEG	NEG	NEG	NEG	NEG	NEG	NEG	NEG	NEG	NEG	POS	NEG
GKP138-52: in silico prediction	POS	POS	POS	NEG	POS	POS	POS	POS	NEG	NEG	NEG	NEG	NEG	NEG	NEG	NEG	NEG	NEG	POS	NEG
GKP138-78: in silico prediction	POS	POS	POS	NEG	POS	POS	POS	POS	NEG	NEG	NEG	NEG	NEG	NEG	NEG	NEG	NEG	NEG	POS	NEG
GKP136-4: in silico prediction	POS	POS	POS	NEG	POS	POS	POS	POS	NEG	NEG	NEG	NEG	NEG	NEG	NEG	NEG	NEG	NEG	POS	NEG
ZTA09_03668_9_HSA: in silico prediction	POS	POS	POS	NEG	POS	POS	POS	POS	NEG	NEG	NEG	NEG	NEG	NEG	NEG	NEG	NEG	NEG	POS	NEG
GKP136-58: in silico prediction	POS	POS	POS	NEG	POS	POS	POS	POS	NEG	NEG	NEG	NEG	NEG	NEG	NEG	NEG	NEG	NEG	POS	NEG
004_0004_23k: in silico prediction	POS	POS	POS	NEG	POS	POS	POS	POS	NEG	NEG	NEG	NEG	NEG	NEG	NEG	NEG	NEG	NEG	POS	NEG
BA06_02038: in silico prediction	POS	POS	POS	NEG	POS	POS	POS	POS	NEG	NEG	NEG	NEG	NEG	NEG	NEG	NEG	NEG	NEG	POS	NEG
GKP138-4: in silico prediction	POS	POS	POS	NEG	POS	POS	POS	POS	NEG	NEG	NEG	NEG	NEG	NEG	NEG	NEG	NEG	NEG	POS	NEG
ZTA10_02421_9_HSA: in silico prediction	POS	POS	POS	NEG	POS	POS	POS	POS	NEG	NEG	NEG	NEG	NEG	NEG	NEG	NEG	NEG	NEG	POS	NEG
GKP136-62: in silico prediction	POS	POS	POS	NEG	POS	POS	POS	POS	NEG	NEG	NEG	NEG	NEG	NEG	NEG	NEG	NEG	NEG	POS	NEG
LGA251: in silico prediction	POS	POS	POS	NEG	POS	POS	POS	POS	NEG	NEG	NEG	NEG	NEG	NEG	NEG	NEG	NEG	NEG	POS	NEG
UK_NCTC13552: in silico prediction	POS	POS	POS	NEG	POS	POS	POS	POS	NEG	NEG	NEG	NEG	NEG	NEG	NEG	NEG	NEG	NEG	POS	NEG
GKP138-71: in silico prediction	POS	POS	POS	NEG	POS	POS	POS	POS	NEG	NEG	NEG	NEG	NEG	NEG	NEG	NEG	NEG	NEG	POS	NEG
cat-mandible-2013: in silico prediction	POS	POS	POS	NEG	POS	POS	POS	POS	NEG	NEG	NEG	NEG	NEG	NEG	NEG	NEG	NEG	NEG	POS	NEG
10_7550_D: in silico prediction	POS	POS	POS	NEG	POS	POS	POS	POS	NEG	NEG	NEG	NEG	NEG	NEG	NEG	NEG	NEG	NEG	POS	NEG
H114440275_A: in silico prediction	POS	POS	POS	NEG	POS	POS	POS	POS	NEG	NEG	NEG	NEG	NEG	NEG	NEG	NEG	NEG	NEG	POS	NEG

STRAIN / ISOLATE	VIRULENCE : PROTEASES										VIRULENCE : STAPHYLOCOCCAL SUPERANTIGEN/ENTEROTOXIN-LIKE GENES (SET/SSL)								
	aur			splA	splB	splE	sspA	sspB	sspP		setC / selX	ssl01						ssl02	
	aur (cons)	aur (Other than MRSA252)	aur (MRSA252)						sspP (cons)	sspP (other than ST93)		ssl01/set6 (COL)	ssl01/set6 (Mu50+ N315)	ssl01/set6 (MW2+ MSSA476)	ssl01/set6 (MRSA252)	ssl01/set6 (RF122)	ssl01/set6 (other alleles)	ssl02/set7	ssl02/set7 (MRSA252)
	aureolysin			serin- protease A	serin- protease B	serin- protease E	glutamylendopeptidase	Staphopain B, protease	Staphopain A (Staphylopain A), protease		Staphyl. exotoxin-like protein	Staphylococcal superantigen-like protein 1						Staphylococcal superantigen-like protein 2	
V40-124624	POS	POS	NEG	POS	POS	POS	POS	POS	POS	POS	POS	NEG	NEG	NEG	NEG	POS	NEG	POS	NEG
V40-124624: in silico prediction	POS	POS	NEG	POS	POS	POS	POS	POS	POS	POS	POS	NEG	NEG	NEG	NEG	POS	NEG	POS	NEG
SA-120: in silico prediction	POS	POS	NEG	POS	POS	POS	POS	POS	POS	POS	POS	NEG	NEG	NEG	NEG	POS	NEG	POS	AMB
61908: in silico prediction	POS	POS	NEG	POS	POS	POS	POS	POS	POS	POS	POS	NEG	NEG	NEG	NEG	POS	NEG	POS	AMB
M1790_98_1: in silico prediction	POS	POS	NEG	POS	POS	NEG	POS	POS	POS	POS	POS	NEG	NEG	NEG	NEG	POS	NEG	POS	AMB
GKP138-13: in silico prediction	POS	POS	NEG	POS	POS	POS	POS	POS	POS	POS	POS	NEG	NEG	NEG	NEG	POS	NEG	POS	AMB
GKP138-2: in silico prediction	POS	POS	NEG	POS	POS	POS	POS	POS	POS	POS	POS	NEG	NEG	NEG	NEG	POS	NEG	POS	AMB
GKP138-31: in silico prediction	POS	POS	NEG	POS	POS	POS	POS	POS	POS	POS	POS	NEG	NEG	NEG	NEG	POS	NEG	POS	AMB
GKP138-33: in silico prediction	POS	POS	NEG	POS	POS	POS	POS	POS	POS	POS	POS	NEG	NEG	NEG	NEG	POS	NEG	POS	AMB
GKP138-52: in silico prediction	POS	POS	NEG	POS	POS	POS	POS	POS	POS	POS	POS	NEG	NEG	NEG	NEG	POS	NEG	POS	AMB
GKP138-78: in silico prediction	POS	POS	NEG	POS	POS	POS	POS	POS	POS	POS	POS	NEG	NEG	NEG	NEG	POS	NEG	POS	AMB
GKP136-4: in silico prediction	POS	POS	NEG	POS	POS	POS	POS	POS	POS	POS	NEG	NEG	NEG	NEG	NEG	POS	NEG	POS	AMB
ZTA09_03668_9_HSA: in silico prediction	POS	POS	NEG	POS	POS	POS	POS	POS	POS	POS	POS	NEG	NEG	NEG	NEG	POS	NEG	POS	AMB
GKP136-58: in silico prediction	POS	POS	NEG	POS	POS	POS	POS	POS	POS	POS	POS	NEG	NEG	NEG	NEG	POS	NEG	POS	AMB
004_0004_23k: in silico prediction	POS	POS	NEG	POS	POS	POS	POS	POS	POS	POS	POS	NEG	NEG	NEG	NEG	POS	NEG	POS	AMB
BA06_02038: in silico prediction	POS	POS	NEG	POS	POS	POS	POS	POS	POS	POS	POS	NEG	NEG	NEG	NEG	POS	NEG	POS	AMB
GKP138-4: in silico prediction	POS	POS	NEG	POS	POS	POS	POS	POS	POS	POS	POS	NEG	NEG	NEG	NEG	POS	NEG	POS	AMB
ZTA10_02421_9_HSA: in silico prediction	POS	POS	NEG	POS	POS	NEG	POS	POS	POS	POS	POS	NEG	NEG	NEG	NEG	POS	NEG	POS	AMB
GKP136-62: in silico prediction	POS	POS	NEG	POS	POS	POS	POS	POS	POS	POS	POS	NEG	NEG	NEG	NEG	POS	NEG	POS	AMB
LGA251: in silico prediction	POS	POS	NEG	POS	POS	POS	POS	POS	POS	POS	POS	NEG	NEG	NEG	NEG	POS	NEG	POS	AMB
UK_NCTC13552: in silico prediction	POS	POS	NEG	POS	POS	POS	POS	POS	POS	POS	POS	NEG	NEG	NEG	NEG	POS	NEG	POS	NEG
GKP138-71: in silico prediction	POS	POS	NEG	POS	POS	POS	POS	POS	POS	POS	POS	NEG	NEG	NEG	NEG	POS	NEG	POS	AMB
cat-mandible-2013: in silico prediction	POS	POS	NEG	POS	POS	POS	POS	POS	POS	POS	POS	NEG	NEG	NEG	NEG	POS	NEG	POS	AMB
10_7550_D: in silico prediction	POS	POS	NEG	POS	POS	POS	POS	POS	POS	POS	POS	NEG	NEG	NEG	NEG	POS	NEG	POS	AMB
H114440275_A: in silico prediction	POS	POS	NEG	POS	POS	POS	POS	POS	POS	POS	POS	NEG	NEG	NEG	NEG	POS	NEG	POS	AMB

STRAIN / ISOLATE	VIRULENCE : STAPHYLOCOCCAL SUPERANTIGEN/ENTEROTOXIN-LIKE GENES (SET/SSL)																VIRULENCE : SET/SSL GENES				
	ssl03		ssl04		ssl05			ssl06		ssl07			ssl08	ssl09		ssl10			ssl11			
	ssl03/set8	ssl03/set8 (MRSA252, SAR0424)	ssl04/set9	ssl04/set9 (MRSA252, SAR0425)	ssl05/set3	ssl05/set3 (RF122, probe-611)	ssl05/set3 (MRSA252)	ssl06/set21	ssl06 (NCTC8325+ MW2)	ssl07/set1	ssl07/set1 (MRSA252)	ssl07/set1 (AF188836)		ssl09/set5_ probes 1&2	ssl09/set5 (MRSA252)	ssl10/set4	ssl10 (RF122)	ssl10/set4 (MRSA252)	ssl11/set2 (COL)	ssl11/set2 (Mu50+ N315)	ssl11/set2 (MW2+ MSSA476)	ssl11/set2 (MRSA252)
	Staphylococcal superantigen-like protein 3		Staphylococcal superantigen-like protein 4		Staphylococcal superantigen-like protein 5			Staphylococcal superantigen-like protein 6		Staphylococcal superantigen-like protein 7			Staphylococcal superantigen-like protein 8	Staphylococcal superantigen-like protein 9		Staphylococcal superantigen-like protein 10			Staphylococcal superantigene-like protein 11			
V40-124624	NEG	NEG	POS	NEG	NEG	POS	NEG	NEG	NEG	POS	AMB	NEG	POS	NEG	NEG	AMB	POS	NEG	NEG	NEG	NEG	NEG
V40-124624: in silico prediction	POS	NEG	POS	NEG	NEG	POS	NEG	NEG	NEG	POS	NEG	NEG	POS	NEG	NEG	NEG	AMB	NEG	NEG	NEG	NEG	NEG
SA-120: in silico prediction	POS	NEG	POS	NEG	NEG	POS	NEG	NEG	NEG	POS	NEG	NEG	POS	POS	NEG	NEG	AMB	NEG	NEG	NEG	NEG	NEG
61908: in silico prediction	POS	NEG	POS	NEG	NEG	POS	NEG	NEG	NEG	POS	NEG	NEG	POS	POS	NEG	NEG	AMB	NEG	NEG	NEG	NEG	NEG
M1790_98_1: in silico prediction	POS	NEG	POS	NEG	NEG	POS	NEG	NEG	NEG	POS	NEG	NEG	POS	POS	NEG	NEG	AMB	NEG	NEG	NEG	NEG	NEG
GKP138-13: in silico prediction	POS	NEG	POS	NEG	NEG	POS	NEG	NEG	NEG	POS	NEG	NEG	POS	POS	NEG	NEG	AMB	NEG	NEG	NEG	NEG	NEG
GKP138-2: in silico prediction	POS	NEG	POS	NEG	NEG	POS	NEG	NEG	NEG	POS	NEG	NEG	POS	POS	NEG	NEG	AMB	NEG	NEG	NEG	NEG	NEG
GKP138-31: in silico prediction	POS	NEG	POS	NEG	NEG	POS	NEG	NEG	NEG	POS	NEG	NEG	POS	POS	NEG	NEG	AMB	NEG	NEG	NEG	NEG	NEG
GKP138-33: in silico prediction	POS	NEG	POS	NEG	NEG	POS	NEG	NEG	NEG	POS	NEG	NEG	POS	POS	NEG	NEG	AMB	NEG	NEG	NEG	NEG	NEG
GKP138-52: in silico prediction	POS	NEG	POS	NEG	NEG	POS	NEG	NEG	NEG	POS	NEG	NEG	POS	POS	NEG	NEG	AMB	NEG	NEG	NEG	NEG	NEG
GKP138-78: in silico prediction	POS	NEG	POS	NEG	NEG	POS	NEG	NEG	NEG	POS	NEG	NEG	POS	POS	NEG	NEG	AMB	NEG	NEG	NEG	NEG	NEG
GKP136-4: in silico prediction	POS	NEG	POS	NEG	NEG	POS	NEG	NEG	NEG	POS	NEG	NEG	POS	POS	NEG	NEG	AMB	NEG	NEG	NEG	NEG	NEG
ZTA09_03668_9_HSA: in silico prediction	POS	NEG	POS	NEG	NEG	POS	NEG	NEG	NEG	POS	NEG	NEG	POS	POS	NEG	NEG	AMB	NEG	NEG	NEG	NEG	NEG
GKP136-58: in silico prediction	POS	NEG	POS	NEG	NEG	POS	NEG	NEG	NEG	POS	NEG	NEG	POS	POS	NEG	NEG	AMB	NEG	NEG	NEG	NEG	NEG
004_0004_23k: in silico prediction	POS	NEG	POS	NEG	NEG	POS	NEG	NEG	NEG	POS	NEG	NEG	POS	POS	NEG	NEG	AMB	NEG	NEG	NEG	NEG	NEG
BA06_02038: in silico prediction	POS	NEG	POS	NEG	NEG	POS	NEG	NEG	NEG	POS	NEG	NEG	POS	POS	NEG	NEG	AMB	NEG	NEG	NEG	NEG	NEG
GKP138-4: in silico prediction	POS	NEG	POS	NEG	NEG	POS	NEG	NEG	NEG	POS	NEG	NEG	POS	POS	NEG	NEG	AMB	NEG	NEG	NEG	NEG	NEG
ZTA10_02421_9_HSA: in silico prediction	POS	NEG	POS	NEG	NEG	POS	NEG	NEG	NEG	POS	NEG	NEG	POS	POS	NEG	NEG	AMB	NEG	NEG	NEG	NEG	NEG
GKP136-62: in silico prediction	POS	NEG	POS	NEG	NEG	POS	NEG	NEG	NEG	POS	NEG	NEG	POS	POS	NEG	NEG	AMB	NEG	NEG	NEG	NEG	NEG
LGA251: in silico prediction	POS	NEG	POS	NEG	NEG	POS	NEG	NEG	NEG	POS	NEG	NEG	POS	POS	NEG	NEG	AMB	NEG	NEG	NEG	NEG	NEG
UK_NCTC13552: in silico prediction	POS	NEG	POS	NEG	NEG	POS	NEG	NEG	NEG	POS	NEG	NEG	POS	POS	NEG	NEG	AMB	NEG	NEG	NEG	NEG	NEG
GKP138-71: in silico prediction	POS	NEG	POS	NEG	NEG	POS	NEG	NEG	NEG	POS	NEG	NEG	POS	POS	NEG	NEG	AMB	NEG	NEG	NEG	NEG	NEG
cat-mandible-2013: in silico prediction	POS	NEG	POS	NEG	NEG	POS	NEG	NEG	NEG	POS	NEG	NEG	POS	POS	NEG	NEG	AMB	NEG	NEG	NEG	NEG	NEG
10_7550_D: in silico prediction	POS	NEG	POS	NEG	NEG	POS	NEG	NEG	NEG	POS	NEG	NEG	POS	POS	NEG	NEG	AMB	NEG	NEG	NEG	NEG	NEG
H114440275_A: in silico prediction	POS	NEG	POS	NEG	NEG	POS	NEG	NEG	NEG	POS	NEG	NEG	POS	POS	NEG	NEG	AMB	NEG	NEG	NEG	NEG	NEG

STRAIN / ISOLATE	VIRULENCE : SET/SSL GENES																		
	setB3		setB2		setB1	Capsule type 1			Capsule type 5			Capsule type 8				icaA	icaC	icaD	bap
	setB3	setB3 (MRSA252)	setB2	setB2 (MRSA252)		capH1	capJ1	capK1	capH5	capJ5	capK5	capH8	capI8	capJ8	capK8				
	Staphylococcal exotoxin-like protein, second locus					capsular poly-saccharide synthesis enzyme	O-antigen polymerase	capsular poly-saccharide biosyn-thesis protein	capsular poly-saccharide synthesis enzyme	O-antigen polymerase	capsular poly-saccharide biosyn-thesis protein	capsular poly-saccharide synthesis enzyme	capsular poly-saccharide biosyn-thesis protein	O-antigen poly-merase	capsular poly-saccharide biosyn-thesis protein	intercellular adhesion protein A	intercellular adhesion protein C	biofilm PIA synthesis protein D	Surface protein involved in biofilm formation
V40-124624	POS	NEG	POS	NEG	POS	NEG	NEG	NEG	POS	POS	POS	NEG	NEG	NEG	NEG	POS	POS	POS	NEG
V40-124624: in silico prediction	POS	NEG	POS	NEG	POS	NEG	NEG	NEG	POS	POS	POS	NEG	NEG	NEG	NEG	POS	POS	POS	NEG
SA-120: in silico prediction	POS	NEG	POS	NEG	POS	NEG	NEG	NEG	POS	POS	POS	NEG	NEG	NEG	NEG	POS	POS	POS	NEG
61908: in silico prediction	POS	NEG	POS	NEG	POS	NEG	NEG	NEG	POS	POS	POS	NEG	NEG	NEG	NEG	POS	POS	POS	NEG
M1790_98_1: in silico prediction	POS	NEG	POS	NEG	POS	NEG	NEG	NEG	POS	POS	POS	NEG	NEG	NEG	NEG	POS	POS	POS	NEG
GKP138-13: in silico prediction	POS	NEG	POS	NEG	POS	NEG	NEG	NEG	POS	POS	POS	NEG	NEG	NEG	NEG	POS	POS	POS	NEG
GKP138-2: in silico prediction	NEG	NEG	NEG	NEG	NEG	NEG	NEG	NEG	POS	POS	POS	NEG	NEG	NEG	NEG	POS	POS	POS	NEG
GKP138-31: in silico prediction	POS	NEG	POS	NEG	POS	NEG	NEG	NEG	POS	POS	POS	NEG	NEG	NEG	NEG	POS	POS	POS	NEG
GKP138-33: in silico prediction	POS	NEG	POS	NEG	POS	NEG	NEG	NEG	POS	POS	POS	NEG	NEG	NEG	NEG	POS	POS	POS	NEG
GKP138-52: in silico prediction	POS	NEG	POS	NEG	POS	NEG	NEG	NEG	POS	POS	POS	NEG	NEG	NEG	NEG	POS	POS	POS	NEG
GKP138-78: in silico prediction	POS	NEG	POS	NEG	POS	NEG	NEG	NEG	POS	POS	POS	NEG	NEG	NEG	NEG	POS	POS	POS	NEG
GKP136-4: in silico prediction	POS	NEG	POS	NEG	POS	NEG	NEG	NEG	POS	POS	POS	NEG	NEG	NEG	NEG	POS	POS	POS	NEG
ZTA09_03668_9_HSA: in silico prediction	POS	NEG	POS	NEG	POS	NEG	NEG	NEG	POS	POS	POS	NEG	NEG	NEG	NEG	POS	POS	POS	NEG
GKP136-58: in silico prediction	POS	NEG	POS	NEG	POS	NEG	NEG	NEG	POS	POS	POS	NEG	NEG	NEG	NEG	POS	POS	POS	NEG
004_0004_23k: in silico prediction	POS	NEG	POS	NEG	POS	NEG	NEG	NEG	POS	POS	POS	NEG	NEG	NEG	NEG	POS	POS	POS	NEG
BA06_02038: in silico prediction	POS	NEG	POS	NEG	POS	NEG	NEG	NEG	POS	POS	POS	NEG	NEG	NEG	NEG	POS	POS	POS	NEG
GKP138-4: in silico prediction	POS	NEG	POS	NEG	POS	NEG	NEG	NEG	POS	POS	POS	NEG	NEG	NEG	NEG	POS	POS	POS	NEG
ZTA10_02421_9_HSA: in silico prediction	POS	NEG	POS	NEG	POS	NEG	NEG	NEG	POS	POS	POS	NEG	NEG	NEG	NEG	POS	POS	POS	NEG
GKP136-62: in silico prediction	POS	NEG	POS	NEG	POS	NEG	NEG	NEG	POS	POS	POS	NEG	NEG	NEG	NEG	POS	POS	POS	NEG
LGA251: in silico prediction	POS	NEG	POS	NEG	POS	NEG	NEG	NEG	POS	POS	POS	NEG	NEG	NEG	NEG	POS	POS	POS	NEG
UK_NCTC13552: in silico prediction	POS	NEG	POS	NEG	POS	NEG	NEG	NEG	POS	POS	POS	NEG	NEG	NEG	NEG	POS	POS	POS	NEG
GKP138-71: in silico prediction	POS	NEG	POS	NEG	POS	NEG	NEG	NEG	POS	POS	POS	NEG	NEG	NEG	NEG	POS	POS	POS	NEG
cat-mandible-2013: in silico prediction	POS	NEG	POS	NEG	POS	NEG	NEG	NEG	POS	POS	POS	NEG	NEG	NEG	NEG	POS	POS	POS	NEG
10_7550_D: in silico prediction	POS	NEG	POS	NEG	POS	NEG	NEG	NEG	POS	POS	POS	NEG	NEG	NEG	NEG	POS	POS	POS	NEG
H114440275_A: in silico prediction	POS	NEG	POS	NEG	POS	NEG	NEG	NEG	POS	POS	POS	NEG	NEG	NEG	NEG	POS	POS	POS	NEG

STRAIN / ISOLATE	ADHAESION FACTORS / GENES ENCODING MICROBIAL SURFACE COMPONENTS RECOGNIZING ADHESIVE MATRIX MOLECULES (MSCRAMM GENES)																	
	bbp							clfA					clfB					cna
	bbp	bbp (cons)	bbp (COL+MW2)	bbp (MRSA252)	bbp (Mu50)	bbp (RF122)	bbp (ST45)	clfA	clfA (cons)	clfA (COL+RF122)	clfA (MRSA252)	clfA (Mu50+MW2)	clfB	clfB (cons)	clfB (COL+Mu50)	clfB (MW2)	clfB (RF122)	
	Bone sialoprotein-binding protein							Clumping factor A					Clumping factor B					Collagen-binding adhesin
V40-124624	POS	POS	NEG	NEG	NEG	NEG	NEG	POS	POS	NEG	NEG	POS	POS	POS	NEG	NEG	AMB	NEG
V40-124624: in silico prediction	POS	POS	NEG	NEG	NEG	NEG	NEG	POS	POS	NEG	NEG	POS	POS	POS	NEG	NEG	NEG	AMB
SA-120: in silico prediction	POS	POS	NEG	NEG	NEG	NEG	NEG	POS	POS	NEG	NEG	POS	POS	POS	NEG	NEG	NEG	AMB
61908: in silico prediction	POS	POS	NEG	NEG	NEG	NEG	NEG	POS	POS	NEG	NEG	POS	POS	POS	NEG	NEG	NEG	AMB
M1790_98_1: in silico prediction	POS	POS	NEG	NEG	NEG	NEG	NEG	POS	POS	NEG	NEG	POS	POS	POS	NEG	NEG	NEG	AMB
GKP138-13: in silico prediction	POS	POS	NEG	NEG	NEG	NEG	NEG	POS	POS	NEG	NEG	POS	POS	POS	NEG	NEG	NEG	AMB
GKP138-2: in silico prediction	POS	POS	NEG	NEG	NEG	NEG	NEG	POS	POS	NEG	NEG	POS	POS	POS	NEG	NEG	NEG	AMB
GKP138-31: in silico prediction	POS	POS	NEG	NEG	NEG	NEG	NEG	POS	POS	NEG	NEG	POS	POS	POS	NEG	NEG	NEG	AMB
GKP138-33: in silico prediction	POS	POS	NEG	NEG	NEG	NEG	NEG	POS	POS	NEG	NEG	POS	POS	POS	NEG	NEG	NEG	AMB
GKP138-52: in silico prediction	POS	POS	NEG	NEG	NEG	NEG	NEG	POS	POS	NEG	NEG	POS	POS	POS	NEG	NEG	NEG	AMB
GKP138-78: in silico prediction	POS	POS	NEG	NEG	NEG	NEG	NEG	POS	POS	NEG	NEG	POS	POS	POS	NEG	NEG	NEG	AMB
GKP136-4: in silico prediction	POS	POS	NEG	NEG	NEG	NEG	NEG	POS	POS	NEG	NEG	POS	POS	POS	NEG	NEG	NEG	AMB
ZTA09_03668_9_HSA: in silico prediction	POS	POS	NEG	NEG	NEG	NEG	NEG	POS	POS	NEG	NEG	POS	POS	POS	NEG	NEG	NEG	AMB
GKP136-58: in silico prediction	POS	POS	NEG	NEG	NEG	NEG	NEG	POS	POS	NEG	NEG	POS	POS	POS	NEG	NEG	NEG	AMB
004_0004_23k: in silico prediction	POS	POS	NEG	NEG	NEG	NEG	NEG	POS	POS	NEG	NEG	POS	POS	POS	NEG	NEG	NEG	AMB
BA06_02038: in silico prediction	POS	POS	NEG	NEG	NEG	NEG	NEG	POS	POS	NEG	NEG	POS	POS	POS	NEG	NEG	NEG	AMB
GKP138-4: in silico prediction	POS	POS	NEG	NEG	NEG	NEG	NEG	POS	POS	NEG	NEG	POS	POS	POS	NEG	NEG	NEG	AMB
ZTA10_02421_9_HSA: in silico prediction	POS	POS	NEG	NEG	NEG	NEG	NEG	POS	POS	NEG	NEG	POS	POS	POS	NEG	NEG	NEG	AMB
GKP136-62: in silico prediction	POS	POS	NEG	NEG	NEG	NEG	NEG	POS	POS	NEG	NEG	POS	POS	POS	NEG	NEG	NEG	AMB
LGA251: in silico prediction	POS	POS	NEG	NEG	NEG	NEG	NEG	POS	POS	NEG	NEG	POS	POS	POS	NEG	NEG	NEG	AMB
UK_NCTC13552: in silico prediction	POS	POS	NEG	NEG	NEG	NEG	NEG	POS	POS	NEG	NEG	POS	POS	POS	NEG	NEG	NEG	AMB
GKP138-71: in silico prediction	POS	POS	NEG	NEG	NEG	NEG	NEG	POS	POS	NEG	NEG	POS	POS	POS	NEG	NEG	NEG	AMB
cat-mandible-2013: in silico prediction	POS	POS	NEG	NEG	NEG	NEG	NEG	POS	POS	NEG	NEG	POS	POS	POS	NEG	NEG	NEG	AMB
10_7550_D: in silico prediction	POS	POS	NEG	NEG	NEG	NEG	NEG	POS	POS	NEG	NEG	POS	POS	POS	NEG	NEG	NEG	AMB
H114440275_A: in silico prediction	POS	POS	NEG	NEG	NEG	NEG	NEG	POS	POS	NEG	NEG	POS	POS	POS	NEG	NEG	NEG	AMB

STRAIN / ISOLATE	ADHAESION FACTORS / GENES ENCODING MICROBIAL SURFACE COMPONENTS RECOGNIZING ADHESIVE MATRIX MOLECULES (MSCRAMM GENES)														
	ebh	ebpS					eno	efb		fnbA					
	ebh (cons)	ebpS	ebpS_probe 612	ebpS_probe 614	ebpS (01-1111)	ebpS (COL)	eno	efb / fib	efb / fib (MRSA252)	fnbA	fnbA (cons)	fnbA (COL)	fnbA (MRSA252)	fnbA (Mu50+MW2)	fnbA (RF122)
	Cell wall associated fibronectin-binding protein	cell surface elastin binding protein					enolase	fibrinogen binding protein (19 kDa)		fibronectin-binding protein A					
V40-124624	POS	POS	NEG	POS	POS	NEG	POS	POS	NEG	POS	POS	NEG	NEG	NEG	NEG
V40-124624: in silico prediction	POS	POS	NEG	POS	POS	NEG	POS	POS	NEG	POS	POS	NEG	NEG	NEG	NEG
SA-120: in silico prediction	POS	POS	NEG	POS	POS	NEG	POS	POS	NEG	POS	POS	NEG	NEG	NEG	NEG
61908: in silico prediction	POS	POS	NEG	POS	POS	NEG	POS	POS	NEG	POS	POS	NEG	NEG	NEG	NEG
M1790_98_1: in silico prediction	POS	POS	NEG	POS	POS	NEG	POS	POS	NEG	POS	POS	NEG	NEG	NEG	NEG
GKP138-13: in silico prediction	POS	POS	NEG	POS	POS	NEG	POS	POS	NEG	POS	POS	NEG	NEG	NEG	NEG
GKP138-2: in silico prediction	POS	POS	NEG	POS	POS	NEG	POS	POS	NEG	POS	POS	NEG	NEG	NEG	NEG
GKP138-31: in silico prediction	POS	POS	NEG	POS	POS	NEG	POS	POS	NEG	POS	POS	NEG	NEG	NEG	NEG
GKP138-33: in silico prediction	POS	POS	NEG	POS	POS	NEG	POS	POS	NEG	POS	POS	NEG	NEG	NEG	NEG
GKP138-52: in silico prediction	POS	POS	NEG	POS	POS	NEG	POS	POS	NEG	POS	POS	NEG	NEG	NEG	NEG
GKP138-78: in silico prediction	POS	POS	NEG	POS	POS	NEG	POS	POS	NEG	POS	POS	NEG	NEG	NEG	NEG
GKP136-4: in silico prediction	POS	POS	NEG	POS	POS	NEG	POS	POS	NEG	POS	POS	NEG	NEG	NEG	NEG
ZTA09_03668_9_HSA: in silico prediction	POS	POS	NEG	POS	POS	NEG	POS	POS	NEG	POS	POS	NEG	NEG	NEG	NEG
GKP136-58: in silico prediction	POS	POS	NEG	POS	POS	NEG	POS	POS	NEG	POS	POS	NEG	NEG	NEG	NEG
004_0004_23k: in silico prediction	POS	POS	NEG	POS	POS	NEG	POS	POS	NEG	POS	POS	NEG	NEG	NEG	NEG
BA06_02038: in silico prediction	POS	POS	NEG	POS	POS	NEG	POS	POS	NEG	POS	POS	NEG	NEG	NEG	NEG
GKP138-4: in silico prediction	POS	POS	NEG	POS	POS	NEG	POS	POS	NEG	POS	POS	NEG	NEG	NEG	NEG
ZTA10_02421_9_HSA: in silico prediction	POS	POS	NEG	POS	POS	NEG	POS	POS	NEG	POS	POS	NEG	NEG	NEG	NEG
GKP136-62: in silico prediction	POS	POS	NEG	POS	POS	NEG	POS	POS	NEG	POS	POS	NEG	NEG	NEG	NEG
LGA251: in silico prediction	POS	POS	NEG	POS	POS	NEG	POS	POS	NEG	POS	POS	NEG	NEG	NEG	NEG
UK_NCTC13552: in silico prediction	POS	POS	NEG	POS	POS	NEG	POS	POS	NEG	POS	POS	NEG	NEG	NEG	NEG
GKP138-71: in silico prediction	POS	POS	NEG	POS	POS	NEG	POS	POS	NEG	POS	POS	NEG	NEG	NEG	NEG
cat-mandible-2013: in silico prediction	POS	POS	NEG	POS	POS	NEG	POS	POS	NEG	POS	POS	NEG	NEG	NEG	NEG
10_7550_D: in silico prediction	POS	POS	NEG	POS	POS	NEG	POS	POS	NEG	POS	POS	NEG	NEG	NEG	NEG
H114440275_A: in silico prediction	POS	POS	NEG	POS	POS	NEG	POS	POS	NEG	POS	POS	NEG	NEG	NEG	NEG

STRAIN / ISOLATE	ADHAESION FACTORS / GENES ENCODING MICROBIAL SURFACE COMPONENTS RECOGNIZING ADHESIVE MATRIX MOLECULES (MSCRAMM GENES)															
	fnbB							map				sasG				
	fnbB	fnbB (COL)	fnbB (COL+Mu50+MW2)	fnbB (Mu50)	fnbB (MW2)	fnbB (ST15)	fnbB (ST45-2)	map	map (COL)	map (MRSA252)	map (Mu50+MW2)	sasG	sasG (COL+Mu50)	sasG (MW2)	sasG (OtherThan252+122	sasX / sesI
	Fibronectin-binding protein B							Major histocompatibility complex class II analog protein (=Extracellular adherence protein, eap)				Staphylococcus aureus surface protein G				
V40-124624	POS	NEG	POS	AMB	NEG	NEG	NEG	POS	POS	NEG	NEG	NEG	NEG	NEG	NEG	NEG
V40-124624: in silico prediction	POS	NEG	AMB	AMB	NEG	NEG	NEG	POS	POS	NEG	NEG	NEG	NEG	NEG	NEG	NEG
SA-120: in silico prediction	NEG	NEG	NEG	NEG	NEG	NEG	NEG	POS	POS	NEG	NEG	NEG	NEG	NEG	NEG	NEG
61908: in silico prediction	NEG	NEG	NEG	NEG	NEG	NEG	NEG	POS	POS	NEG	NEG	NEG	NEG	NEG	NEG	NEG
M1790_98_1: in silico prediction	POS	NEG	AMB	AMB	NEG	NEG	NEG	POS	POS	NEG	NEG	NEG	NEG	NEG	NEG	NEG
GKP138-13: in silico prediction	POS	NEG	AMB	AMB	NEG	NEG	NEG	POS	POS	NEG	NEG	NEG	NEG	NEG	NEG	NEG
GKP138-2: in silico prediction	POS	NEG	AMB	AMB	NEG	NEG	NEG	POS	POS	NEG	NEG	NEG	NEG	NEG	NEG	NEG
GKP138-31: in silico prediction	POS	NEG	AMB	AMB	NEG	NEG	NEG	POS	POS	NEG	NEG	NEG	NEG	NEG	NEG	NEG
GKP138-33: in silico prediction	POS	NEG	AMB	AMB	NEG	NEG	NEG	POS	POS	NEG	NEG	NEG	NEG	NEG	NEG	NEG
GKP138-52: in silico prediction	POS	NEG	AMB	AMB	NEG	NEG	NEG	POS	POS	NEG	NEG	NEG	NEG	NEG	NEG	NEG
GKP138-78: in silico prediction	POS	NEG	AMB	AMB	NEG	NEG	NEG	POS	POS	NEG	NEG	NEG	NEG	NEG	NEG	NEG
GKP136-4: in silico prediction	NEG	NEG	NEG	NEG	NEG	NEG	NEG	POS	POS	NEG	NEG	NEG	NEG	NEG	NEG	NEG
ZTA09_03668_9_HSA: in silico prediction	POS	NEG	AMB	AMB	NEG	NEG	NEG	POS	POS	NEG	NEG	NEG	NEG	NEG	NEG	NEG
GKP136-58: in silico prediction	POS	NEG	AMB	AMB	NEG	NEG	NEG	POS	POS	NEG	NEG	NEG	NEG	NEG	NEG	NEG
004_0004_23k: in silico prediction	POS	NEG	AMB	AMB	NEG	NEG	NEG	POS	POS	NEG	NEG	NEG	NEG	NEG	NEG	NEG
BA06_02038: in silico prediction	POS	NEG	AMB	AMB	NEG	NEG	NEG	POS	POS	NEG	NEG	NEG	NEG	NEG	NEG	NEG
GKP138-4: in silico prediction	POS	NEG	AMB	AMB	NEG	NEG	NEG	POS	POS	NEG	NEG	NEG	NEG	NEG	NEG	NEG
ZTA10_02421_9_HSA: in silico prediction	POS	NEG	AMB	AMB	NEG	NEG	NEG	POS	POS	NEG	NEG	NEG	NEG	NEG	NEG	NEG
GKP136-62: in silico prediction	POS	NEG	AMB	AMB	NEG	NEG	NEG	POS	POS	NEG	NEG	NEG	NEG	NEG	NEG	NEG
LGA251: in silico prediction	POS	NEG	AMB	AMB	NEG	NEG	NEG	POS	POS	NEG	NEG	NEG	NEG	NEG	NEG	NEG
UK_NCTC13552: in silico prediction	POS	NEG	AMB	AMB	NEG	NEG	NEG	POS	POS	NEG	NEG	NEG	NEG	NEG	NEG	NEG
GKP138-71: in silico prediction	POS	NEG	AMB	AMB	NEG	NEG	NEG	POS	POS	NEG	NEG	NEG	NEG	NEG	NEG	NEG
cat-mandible-2013: in silico prediction	POS	NEG	AMB	AMB	NEG	NEG	NEG	POS	POS	NEG	NEG	NEG	NEG	NEG	NEG	NEG
10_7550_D: in silico prediction	NEG	NEG	NEG	NEG	NEG	NEG	NEG	POS	POS	NEG	NEG	NEG	NEG	NEG	NEG	NEG
H114440275_A: in silico prediction	POS	NEG	AMB	AMB	NEG	NEG	NEG	POS	POS	NEG	NEG	NEG	NEG	NEG	NEG	NEG

STRAIN / ISOLATE	ADHAESION FACTORS / MSCRAMM GENES																
	sdrC						sdrD					vwb					
	sdrC	sdrC (B1)	sdrC (COL)	sdrC (Mu50)	sdrC (MW2+MRSA 252+RF122)	sdrC (OtherThan25 2+RF122)	sdrD	sdrD (cons)	sdrD (COL+MW2)	sdrD (Mu50)	sdrD (other)	vwb	vwb (cons)	vwb (COL+MW2)	vwb (MRSA252)	vwb (Mu50)	vwb (RF122)
	Ser-Asp rich fibrinogen-/bone sialoprotein-binding protein C						Ser-Asp rich fibrinogen-/bone sialoprotein-binding protein D					van Willebrand factor binding protein					
V40-124624	POS	NEG	NEG	NEG	NEG	NEG	POS	POS	NEG	NEG	POS	POS	POS	NEG	NEG	NEG	NEG
V40-124624: in silico prediction	POS	NEG	NEG	NEG	POS	NEG	POS	POS	NEG	NEG	POS	POS	POS	NEG	NEG	NEG	NEG
SA-120: in silico prediction	POS	NEG	NEG	NEG	POS	NEG	POS	POS	NEG	NEG	POS	POS	POS	NEG	NEG	NEG	NEG
61908: in silico prediction	POS	NEG	NEG	NEG	POS	NEG	POS	POS	NEG	NEG	POS	POS	POS	NEG	NEG	NEG	NEG
M1790_98_1: in silico prediction	POS	NEG	NEG	NEG	POS	NEG	POS	POS	NEG	NEG	POS	POS	POS	NEG	NEG	NEG	NEG
GKP138-13: in silico prediction	POS	NEG	NEG	NEG	POS	NEG	POS	POS	NEG	NEG	POS	POS	POS	NEG	NEG	NEG	NEG
GKP138-2: in silico prediction	POS	NEG	NEG	NEG	POS	NEG	POS	POS	NEG	NEG	POS	NEG	NEG	NEG	NEG	NEG	NEG
GKP138-31: in silico prediction	POS	NEG	NEG	NEG	POS	NEG	POS	POS	NEG	NEG	POS	NEG	NEG	NEG	NEG	NEG	NEG
GKP138-33: in silico prediction	POS	NEG	NEG	NEG	POS	NEG	POS	POS	NEG	NEG	POS	POS	POS	NEG	NEG	NEG	NEG
GKP138-52: in silico prediction	POS	NEG	NEG	NEG	POS	NEG	POS	POS	NEG	NEG	POS	POS	POS	NEG	NEG	NEG	NEG
GKP138-78: in silico prediction	POS	NEG	NEG	NEG	POS	NEG	POS	POS	NEG	NEG	POS	POS	POS	NEG	NEG	NEG	NEG
GKP136-4: in silico prediction	POS	NEG	NEG	NEG	POS	NEG	POS	POS	NEG	NEG	POS	POS	POS	NEG	NEG	NEG	NEG
ZTA09_03668_9_HSA: in silico prediction	POS	NEG	NEG	NEG	POS	NEG	NEG	NEG	NEG	NEG	NEG	POS	POS	NEG	NEG	NEG	NEG
GKP136-58: in silico prediction	POS	NEG	NEG	NEG	POS	NEG	POS	POS	NEG	NEG	POS	POS	POS	NEG	NEG	NEG	NEG
004_0004_23k: in silico prediction	POS	NEG	NEG	NEG	POS	NEG	POS	POS	NEG	NEG	POS	POS	POS	NEG	NEG	NEG	NEG
BA06_02038: in silico prediction	POS	NEG	NEG	NEG	POS	NEG	POS	POS	NEG	NEG	POS	POS	POS	NEG	NEG	NEG	NEG
GKP138-4: in silico prediction	POS	NEG	NEG	NEG	POS	NEG	POS	POS	NEG	NEG	POS	POS	POS	NEG	NEG	NEG	NEG
ZTA10_02421_9_HSA: in silico prediction	POS	NEG	NEG	NEG	POS	NEG	POS	POS	NEG	NEG	POS	POS	POS	NEG	NEG	NEG	NEG
GKP136-62: in silico prediction	POS	NEG	NEG	NEG	POS	NEG	POS	POS	NEG	NEG	POS	POS	POS	NEG	NEG	NEG	NEG
LGA251: in silico prediction	POS	NEG	NEG	NEG	POS	NEG	POS	POS	NEG	NEG	POS	POS	POS	NEG	NEG	NEG	NEG
UK_NCTC13552: in silico prediction	POS	NEG	NEG	NEG	POS	NEG	POS	POS	NEG	NEG	POS	POS	POS	NEG	NEG	NEG	NEG
GKP138-71: in silico prediction	POS	NEG	NEG	NEG	POS	NEG	POS	POS	NEG	NEG	POS	POS	POS	NEG	NEG	NEG	NEG
cat-mandible-2013: in silico prediction	POS	NEG	NEG	NEG	POS	NEG	POS	POS	NEG	NEG	POS	POS	POS	NEG	NEG	NEG	NEG
10_7550_D: in silico prediction	POS	NEG	NEG	NEG	POS	NEG	POS	NEG	NEG	NEG	POS	POS	POS	NEG	NEG	NEG	NEG
H114440275_A: in silico prediction	POS	NEG	NEG	NEG	POS	NEG	POS	POS	NEG	NEG	POS	POS	POS	NEG	NEG	NEG	NEG

STRAIN / ISOLATE	IMMUNOD.AG.B		DEFENSIN RESIST.		TRANSFERRIN BINDING PROT			PUTATIVE TRANSPORTER				TYPE I RESTRICTION-MODIFICATION SYSTEM, SINGLE SEQUENCE SPECIFICITY PROTEIN				
	isaB		mprF		isdA			lmrP				hsdS1	hsdS2			
	isaB	isaB (MRSA252)	mprF (COL+MW2)	mprF (Mu50+252)	isdA (cons)	isdA (MRSA252)	isdA (Other Than MRSA252)	lmrP (OtherThanRF 122)	lmrP (OtherThanRF 122)	lmrP (RF122)	lmrP (RF122)	hsdS1-RF122	hsdS2-ST5+ST8	hsdS2-MW2+476	hsdS2-RF122	hsdS2-MRSA252
	immunodominant antigen B		defensin resistance protein		transferrin-binding protein			hypothetical protein, similar to integral membrane protein LmrP				type I site-specific deoxyribo-nuclease subunit, 1st locus	type I site-specific deoxyribonuclease subunit, 2nd locus			
V40-124624	POS	POS	NEG	NEG	POS	NEG	POS	NEG	NEG	POS	POS	NEG	NEG	NEG	NEG	POS
V40-124624: in silico prediction	POS	NEG	NEG	POS	POS	NEG	POS	NEG	NEG	POS	POS	NEG	NEG	NEG	NEG	POS
SA-120: in silico prediction	POS	NEG	NEG	POS	POS	NEG	POS	NEG	NEG	POS	POS	NEG	NEG	NEG	NEG	POS
61908: in silico prediction	POS	NEG	NEG	POS	POS	NEG	POS	NEG	NEG	POS	POS	NEG	NEG	NEG	NEG	POS
M1790_98_1: in silico prediction	POS	NEG	NEG	POS	POS	NEG	POS	NEG	NEG	POS	POS	NEG	NEG	NEG	NEG	POS
GKP138-13: in silico prediction	POS	NEG	NEG	POS	POS	NEG	POS	NEG	NEG	POS	POS	NEG	NEG	NEG	NEG	POS
GKP138-2: in silico prediction	POS	NEG	NEG	POS	POS	NEG	POS	NEG	NEG	POS	POS	NEG	NEG	NEG	NEG	POS
GKP138-31: in silico prediction	POS	NEG	NEG	POS	POS	NEG	POS	NEG	NEG	POS	POS	NEG	NEG	NEG	NEG	POS
GKP138-33: in silico prediction	POS	NEG	NEG	POS	POS	NEG	POS	NEG	NEG	POS	POS	NEG	NEG	NEG	NEG	POS
GKP138-52: in silico prediction	POS	NEG	NEG	POS	POS	NEG	POS	NEG	NEG	POS	POS	NEG	NEG	NEG	NEG	POS
GKP138-78: in silico prediction	POS	NEG	NEG	POS	POS	NEG	POS	NEG	NEG	POS	POS	NEG	NEG	NEG	NEG	POS
GKP136-4: in silico prediction	POS	NEG	NEG	POS	POS	NEG	POS	NEG	NEG	POS	POS	NEG	NEG	NEG	NEG	POS
ZTA09_03668_9_HSA: in silico prediction	POS	NEG	NEG	POS	POS	NEG	POS	NEG	NEG	POS	POS	NEG	NEG	NEG	NEG	POS
GKP136-58: in silico prediction	POS	NEG	NEG	POS	POS	NEG	POS	NEG	NEG	POS	POS	NEG	NEG	NEG	NEG	POS
004_0004_23k: in silico prediction	POS	NEG	NEG	POS	POS	NEG	POS	NEG	NEG	POS	POS	NEG	NEG	NEG	NEG	POS
BA06_02038: in silico prediction	POS	NEG	NEG	POS	POS	NEG	POS	NEG	NEG	POS	POS	NEG	NEG	NEG	NEG	POS
GKP138-4: in silico prediction	POS	NEG	NEG	POS	POS	NEG	POS	NEG	NEG	POS	POS	NEG	NEG	NEG	NEG	POS
ZTA10_02421_9_HSA: in silico prediction	POS	NEG	NEG	POS	POS	NEG	POS	NEG	NEG	POS	POS	NEG	NEG	NEG	NEG	POS
GKP136-62: in silico prediction	POS	NEG	NEG	POS	POS	NEG	POS	NEG	NEG	POS	POS	NEG	NEG	NEG	NEG	POS
LGA251: in silico prediction	POS	NEG	NEG	POS	POS	NEG	POS	NEG	NEG	POS	POS	NEG	NEG	NEG	NEG	POS
UK_NCTC13552: in silico prediction	POS	NEG	NEG	POS	POS	NEG	POS	NEG	NEG	POS	POS	NEG	NEG	NEG	NEG	POS
GKP138-71: in silico prediction	POS	NEG	NEG	POS	POS	NEG	POS	NEG	NEG	POS	POS	NEG	NEG	NEG	NEG	POS
cat-mandible-2013: in silico prediction	POS	NEG	NEG	POS	POS	NEG	POS	NEG	NEG	POS	POS	NEG	NEG	NEG	NEG	POS
10_7550_D: in silico prediction	POS	NEG	NEG	POS	POS	NEG	POS	NEG	NEG	POS	POS	NEG	NEG	NEG	NEG	POS
H114440275_A: in silico prediction	POS	NEG	NEG	POS	POS	NEG	POS	NEG	NEG	POS	POS	NEG	NEG	NEG	NEG	POS

STRAIN / ISOLATE	TYPE I RESTRICTION-MODIFICATION SYSTEM, SINGLE SEQUENCE SPECIFICITY PROTEIN								MISCELLANEOUS GENES							
	hsdS3					hsdSx			ear2 = Q2FXC0	Q2YUB3	Q7A4X2	Q931R4 (CC5, CC15, CC30, CC97, CC188, ST1850)	Q9RL82			Q2G1R6- genomic island / cstB
	hsdS3-AllOtherThan RF122+252	hsdS3-ST8+ST1+RF122	hsdS3-Mu50+N315	hsdS3-CC51+252	hsdS3-MRSA252	hsdSx-CC25	hsdSx-CC15	hsdSx-etd					Q9RL82 (consensus)	Q9RL82 (CC8)	Q9RL82-CC10/361	
	type I site-specific deoxyribonuclease subunit, 3rd locus					type I site-specific deoxyribonuclease subunit, unknown locus			Putative protein	Multidrug resistance transporter	Putative protein	major facilitator superfamily transporter	Putative protein			
V40-124624	NEG	NEG	NEG	NEG	NEG	NEG	POS	POS	NEG	NEG	POS	NEG	POS	POS	NEG	POS
V40-124624: in silico prediction	NEG	NEG	NEG	NEG	NEG	NEG	POS	POS	NEG	NEG	POS	NEG	POS	POS	NEG	POS
SA-120: in silico prediction	NEG	NEG	NEG	NEG	NEG	NEG	POS	POS	NEG	NEG	POS	NEG	POS	POS	NEG	POS
61908: in silico prediction	NEG	NEG	NEG	NEG	NEG	NEG	POS	POS	NEG	NEG	POS	NEG	POS	POS	NEG	POS
M1790_98_1: in silico prediction	NEG	NEG	NEG	NEG	NEG	NEG	POS	POS	NEG	NEG	POS	NEG	POS	POS	NEG	POS
GKP138-13: in silico prediction	NEG	NEG	NEG	NEG	NEG	NEG	POS	POS	NEG	NEG	POS	NEG	POS	POS	NEG	POS
GKP138-2: in silico prediction	NEG	NEG	NEG	NEG	NEG	NEG	POS	POS	NEG	NEG	POS	NEG	POS	POS	NEG	POS
GKP138-31: in silico prediction	NEG	NEG	NEG	NEG	NEG	NEG	POS	POS	NEG	NEG	POS	NEG	POS	POS	NEG	POS
GKP138-33: in silico prediction	NEG	NEG	NEG	NEG	NEG	NEG	POS	POS	NEG	NEG	POS	NEG	POS	POS	NEG	POS
GKP138-52: in silico prediction	NEG	NEG	NEG	NEG	NEG	NEG	POS	POS	NEG	NEG	POS	NEG	POS	POS	NEG	POS
GKP138-78: in silico prediction	NEG	NEG	NEG	NEG	NEG	NEG	POS	POS	NEG	NEG	POS	NEG	POS	POS	NEG	POS
GKP136-4: in silico prediction	NEG	NEG	NEG	NEG	NEG	NEG	POS	POS	NEG	NEG	POS	NEG	POS	POS	NEG	POS
ZTA09_03668_9_HSA: in silico prediction	NEG	NEG	NEG	NEG	NEG	NEG	POS	POS	NEG	NEG	POS	NEG	POS	POS	NEG	POS
GKP136-58: in silico prediction	NEG	NEG	NEG	NEG	NEG	NEG	POS	POS	NEG	NEG	POS	NEG	POS	POS	NEG	POS
004_0004_23k: in silico prediction	NEG	NEG	NEG	NEG	NEG	NEG	POS	POS	NEG	NEG	POS	NEG	POS	POS	NEG	POS
BA06_02038: in silico prediction	NEG	NEG	NEG	NEG	NEG	NEG	POS	POS	NEG	NEG	POS	NEG	POS	POS	NEG	POS
GKP138-4: in silico prediction	NEG	NEG	NEG	NEG	NEG	NEG	POS	POS	NEG	NEG	POS	NEG	POS	POS	NEG	POS
ZTA10_02421_9_HSA: in silico prediction	NEG	NEG	NEG	NEG	NEG	NEG	POS	POS	NEG	NEG	POS	NEG	POS	POS	NEG	POS
GKP136-62: in silico prediction	NEG	NEG	NEG	NEG	NEG	NEG	POS	POS	NEG	NEG	POS	NEG	POS	POS	NEG	POS
LGA251: in silico prediction	NEG	NEG	NEG	NEG	NEG	NEG	POS	POS	NEG	NEG	POS	NEG	POS	POS	NEG	POS
UK_NCTC13552: in silico prediction	NEG	NEG	NEG	NEG	NEG	NEG	POS	POS	NEG	NEG	POS	NEG	POS	POS	NEG	POS
GKP138-71: in silico prediction	NEG	NEG	NEG	NEG	NEG	NEG	POS	POS	NEG	NEG	POS	NEG	POS	POS	NEG	POS
cat-mandible-2013: in silico prediction	NEG	NEG	NEG	NEG	NEG	NEG	POS	POS	NEG	NEG	POS	NEG	POS	POS	NEG	POS
10_7550_D: in silico prediction	NEG	NEG	NEG	NEG	NEG	NEG	POS	POS	NEG	NEG	POS	NEG	POS	POS	NEG	POS
H114440275_A: in silico prediction	NEG	NEG	NEG	NEG	NEG	NEG	POS	POS	NEG	NEG	POS	NEG	POS	POS	NEG	POS

STRAIN / ISOLATE	MISCELLANEOUS GENES: sau: sau3AI	sau: sauUSI	sau: sauRF122	sau: sauSO385	sau96I	G7ZRU6	ycjY	sagD	G7ZTC1: G7ZTC1	G7ZTC1: G7ZTC1-argenteus	sdrM / tetEfflux	Q2YUB3: Q2YUB3 (RF122)	HYALURONATE LYASE: hysA1: hysA1 (MRSA252)	hysA1: hysA1 (MRSA252+RF122) and/or hysA2 (cons)	hysA1: hysA1 (MRSA252+RF122) and/or hysA2 (COL+USA300)	hysA2: hysA2 (All Other Than MRSA252)	hysA2: hysA2 (COL+USA300 +NCTC8325)	hysA2: hysA2 (All Other Than COL+USA300+ NCTC8325)	hysA2: hysA2 (All Other Than COL+USA300+ NCTC8325)	hysA2: hysA2 (MRSA252)
	type II restriction-modification system endonuclease					acetyltransferase, GNAT family, "Argenteus/ST1850-like"	Marker for "Argenteus/ST1850-like", CC12, CC361, CC398	Putative bacteriocin biosynthesis associated protein	TetR family regulator		Multidrug resistance transporter		Hyaluronate lyase, variable first / second locus			Hyaluronate lyase, second locus				
V40-124624	NEG	POS	NEG	NEG	NEG	NEG	NEG	NEG	NEG	NEG	POS	NEG	POS	POS	POS	NEG	POS	POS	POS	NEG
V40-124624: in silico prediction	NEG	POS	NEG	NEG	NEG	NEG	NEG	NEG	NEG	NEG	POS	NEG	POS	POS	POS	NEG	NEG	POS	AMB	NEG
SA-120: in silico prediction	NEG	POS	NEG	NEG	NEG	NEG	NEG	NEG	NEG	NEG	POS	NEG	POS	POS	POS	NEG	NEG	POS	AMB	NEG
61908: in silico prediction	NEG	POS	NEG	NEG	NEG	NEG	NEG	NEG	NEG	NEG	POS	NEG	POS	POS	POS	NEG	NEG	POS	AMB	NEG
M1790_98_1: in silico prediction	NEG	POS	NEG	NEG	NEG	NEG	NEG	NEG	NEG	NEG	POS	NEG	POS	POS	POS	NEG	NEG	POS	AMB	NEG
GKP138-13: in silico prediction	NEG	POS	NEG	NEG	NEG	NEG	NEG	NEG	NEG	NEG	POS	NEG	POS	POS	POS	NEG	NEG	POS	AMB	NEG
GKP138-2: in silico prediction	NEG	POS	NEG	NEG	NEG	NEG	NEG	NEG	NEG	NEG	POS	NEG	POS	POS	POS	NEG	NEG	POS	AMB	NEG
GKP138-31: in silico prediction	NEG	POS	NEG	NEG	NEG	NEG	NEG	NEG	NEG	NEG	POS	NEG	POS	POS	POS	NEG	NEG	POS	AMB	NEG
GKP138-33: in silico prediction	NEG	POS	NEG	NEG	NEG	NEG	NEG	NEG	NEG	NEG	POS	NEG	POS	POS	POS	NEG	NEG	POS	AMB	NEG
GKP138-52: in silico prediction	NEG	POS	NEG	NEG	NEG	NEG	NEG	NEG	NEG	NEG	POS	NEG	POS	POS	POS	NEG	NEG	POS	AMB	NEG
GKP138-78: in silico prediction	NEG	POS	NEG	NEG	NEG	NEG	NEG	NEG	NEG	NEG	POS	NEG	POS	POS	POS	NEG	NEG	POS	AMB	NEG
GKP136-4: in silico prediction	NEG	POS	NEG	NEG	NEG	NEG	NEG	NEG	NEG	NEG	POS	NEG	POS	POS	POS	NEG	NEG	POS	AMB	NEG
ZTA09_03668_9_HSA: in silico prediction	NEG	POS	NEG	NEG	NEG	NEG	NEG	NEG	NEG	NEG	POS	NEG	POS	POS	POS	NEG	NEG	POS	AMB	NEG
GKP136-58: in silico prediction	NEG	POS	NEG	NEG	NEG	NEG	NEG	NEG	NEG	NEG	POS	NEG	POS	POS	POS	NEG	NEG	POS	AMB	NEG
004_0004_23k: in silico prediction	NEG	POS	NEG	NEG	NEG	NEG	NEG	NEG	NEG	NEG	POS	NEG	POS	POS	POS	NEG	NEG	POS	AMB	NEG
BA06_02038: in silico prediction	NEG	POS	NEG	NEG	NEG	NEG	NEG	NEG	NEG	NEG	POS	NEG	POS	POS	POS	NEG	NEG	POS	AMB	NEG
GKP138-4: in silico prediction	NEG	POS	NEG	NEG	NEG	NEG	NEG	NEG	NEG	NEG	POS	NEG	POS	POS	POS	NEG	NEG	POS	AMB	NEG
ZTA10_02421_9_HSA: in silico prediction	NEG	POS	NEG	NEG	NEG	NEG	NEG	NEG	NEG	NEG	POS	NEG	POS	POS	POS	NEG	NEG	POS	AMB	NEG
GKP136-62: in silico prediction	NEG	POS	NEG	NEG	NEG	NEG	NEG	NEG	NEG	NEG	POS	NEG	POS	POS	POS	NEG	NEG	POS	AMB	NEG
LGA251: in silico prediction	NEG	POS	NEG	NEG	NEG	NEG	NEG	NEG	NEG	NEG	POS	NEG	POS	POS	POS	NEG	NEG	POS	AMB	NEG
UK_NCTC13552: in silico prediction	NEG	POS	NEG	NEG	NEG	NEG	NEG	NEG	NEG	NEG	POS	NEG	POS	POS	POS	NEG	NEG	POS	AMB	NEG
GKP138-71: in silico prediction	NEG	POS	NEG	NEG	NEG	NEG	NEG	NEG	NEG	NEG	POS	NEG	POS	POS	POS	NEG	NEG	POS	AMB	NEG
cat-mandible-2013: in silico prediction	NEG	POS	NEG	NEG	NEG	NEG	NEG	NEG	NEG	NEG	POS	NEG	POS	POS	POS	NEG	NEG	POS	AMB	NEG
10_7550_D: in silico prediction	NEG	POS	NEG	NEG	NEG	NEG	NEG	NEG	NEG	NEG	POS	NEG	POS	POS	POS	NEG	NEG	POS	AMB	NEG
H114440275_A: in silico prediction	NEG	POS	NEG	NEG	NEG	NEG	NEG	NEG	NEG	NEG	POS	NEG	POS	POS	POS	NEG	NEG	POS	AMB	NEG
